# Use of NON-PARAMETRIC Item Response Theory to develop a shortened version of the Positive and Negative Syndrome Scale (PANSS)

**DOI:** 10.1186/1471-244X-11-178

**Published:** 2011-11-16

**Authors:** Anzalee Khan, Charles Lewis, Jean-Pierre Lindenmayer

**Affiliations:** 1 Fordham University, Department of Psychometrics, Bronx, NY, USA; 2 ProPhase, LLC, New York, NY, USA; 3 New York University, School of Medicine, New York, NY, USA; 4 Nathan S. Kline Institute for Psychiatric Research, Orangeburg, NY, USA; 5 Manhattan Psychiatric Center, Wards Island, NY, USA; 6 Educational Testing Services, ETS, Princeton, NJ, USA

## Abstract

**Background:**

Nonparametric item response theory (IRT) was used to examine (a) the performance of the 30 Positive and Negative Syndrome Scale (PANSS) items and their options ((levels of severity), (b) the effectiveness of various subscales to discriminate among differences in symptom severity, and (c) the development of an abbreviated PANSS (Mini-PANSS) based on IRT and a method to link scores to the original PANSS.

**Methods:**

Baseline PANSS scores from 7,187 patients with Schizophrenia or Schizoaffective disorder who were enrolled between 1995 and 2005 in psychopharmacology trials were obtained. Option characteristic curves (OCCs) and Item Characteristic Curves (ICCs) were constructed to examine the probability of rating each of seven options within each of 30 PANSS items as a function of subscale severity, and summed-score linking was applied to items selected for the Mini-PANSS.

**Results:**

The majority of items forming the Positive and Negative subscales (i.e. 19 items) performed very well and discriminate better along symptom severity compared to the General Psychopathology subscale. Six of the seven Positive Symptom items, six of the seven Negative Symptom items, and seven out of the 16 General Psychopathology items were retained for inclusion in the Mini-PANSS. Summed score linking and linear interpolation was able to produce a translation table for comparing total subscale scores of the Mini-PANSS to total subscale scores on the original PANSS. Results show scores on the subscales of the Mini-PANSS can be linked to scores on the original PANSS subscales, with very little bias.

**Conclusions:**

The study demonstrated the utility of non-parametric IRT in examining the item properties of the PANSS and to allow selection of items for an abbreviated PANSS scale. The comparisons between the 30-item PANSS and the Mini-PANSS revealed that the shorter version is comparable to the 30-item PANSS, but when applying IRT, the Mini-PANSS is also a good indicator of illness severity.

## Background

One of the most widely used measures of psychopathology of schizophrenia in clinical research is the Positive and Negative Syndrome Scale (PANSS) [1,2]. The 30-item PANSS was developed originally for typological and dimensional assessment of patients with schizophrenia [1] and was conceived as an operationalized, change-sensitive instrument that offers balanced representation of positive and negative symptoms and estimates their relationship to one another and to global psychopathology. It consists of three subscales measuring the severity of (a) Positive Symptoms (seven items), (b) Negative Symptoms (seven items), and (c) General Psychopathology (16 items). The PANSS is typically administered by trained clinicians who evaluate patients' current severity level on each item by rating one of seven options (scores) representing increasing levels of severity. The administration generally takes 30 to 60 minutes [1,3], depending on the patient's level of cooperation and severity of symptoms. The PANSS has demonstrated high internal reliability [4,5], good construct validity [4], and excellent sensitivity to change in both short term [6] and long term trials [7]. However, despite extensive psychometric research on the PANSS, until a recent Item Response Analysis [IRT; [8]], it was unclear how individual PANSS items differ in their usefulness in assessing the total severity of symptoms.

Studies examining the psychometric properties of the PANSS have focused on estimates of scale reliability, validity, and factor analysis using methods from Classical Test Theory [CTT; [9]]. These methods rely primarily on omnibus statistics that average across levels of individual variation. Commonly used reliability statistics (e.g., coefficient alpha) may obscure the fact that scale reliability is likely to vary across different levels of severity being measured [10]. Most important, CTT methods cannot weigh the quality of a scale as a function of different levels of psychopathology in the measured disorder.

For unidimensional scales consisting of two or more items with ordered categorical response choices, IRT is a very efficient statistical technique for item selection and score estimation [[11,12] and [13]]. Methods based on IRT provide significant improvements over CTT, as they model the relation between item responses and symptom severity directly, quantifying how the performance of individual items and options (e.g., for PANSS, severity levels range from one to seven) change as a function of overall symptom severity. As schizophrenia is a multidimensional disorder consisting of various symptoms clusters, IRT can be used to test each unidimensional subscale of the PANSS (i.e., Positive Symptoms, Negative Symptoms, and General Psychopathology). IRT analyses can provide unique and relevant information on (a) how well a set of item options assess the entire continuum of symptom severity, (b) whether scores assigned to individual item options are appropriate for measuring a particular trait or symptom, and (c) how well individual items or subscales are connected to the underlying construct and discriminate among individual differences in symptom severity (see Santor and Ramsay [14] for an overview).

IRT can be used to select the most useful items for a shortened scale, and to develop a scoring algorithm that predicts the total score on the full scale [15,16]. Alternatively, previous IRT analysis of the PANSS [8] identified some items that might be further improved for measuring individual severity differences. The analyses showed that 18 of the 30 PANSS items performed well and identified key areas for improvement in items and options within specific subscales. These findings [8] also suggest that the Positive and Negative Symptoms subscales were more sensitive to change than the overall PANSS total score and, thus, may constitute a "Mini-PANSS" that may be more reliable, require shorter time to administer, and possibly reduce sample sizes needed for future research. Additionally, a more recent IRT by Levine and colleagues [17] showed that the PANSS item ratings discriminated symptom severity best for the negative symptoms, have an excess of "Severe" and "Extremely severe" rating options, and assessments are more reliable at medium than very low or high levels of symptom severity.

The present study used IRT to evaluate the PANSS for use in assessing psychopathology in schizophrenia by (a) examining and characterizing the performance of individual items from the PANSS at both the option (severity) and item (symptom) levels and identified areas for improvement of the PANSS scale, by (b) examining the ability of the three PANSS subscales to discriminate among individual difference in illness severity, by (c) selecting the best performing items to be included in a briefer version of the PANSS and by (d) constructing scoring algorithms using a summed score linking technique to directly compare results obtained with the shortened scale to those of the original PANSS scale.

## Methods

### Data

Data was provided for 7,348 patients who met DSM-IV criteria for schizophrenia or schizoaffective disorder, who were enrolled between 1995 and 2005 in one of 16 randomized, double-blind clinical trials comparing risperidone, risperidone depot or paliperidone to other antipsychotic drugs (e.g., haloperidol, olanzapine) or placebo. All studies were carried out in accordance with the latest version of the Declaration of Helsinki. Study procedures were reviewed by the respective ethics committees and informed consent obtained after the procedures was fully explained.

Data analysis included baseline PANSS item scores from 7,187 patients. Table 1 shows the total of number of patients who were removed from the analyses due to diagnoses (other than schizophrenia or schizoaffective disorder (0.04% - 1.09%)) and missing PANSS item scores (0.03%); the mean age, the gender and mean PANSS total score of patients who were removed from each diagnoses group is also presented. The low number of patients excluded assures that analyses would not be compromised by excluding these patients.

**Table 1:** Data Removed from Final Dataset (n = 7,348)

Reason for Removal	Number of cases removed	Percent of cases removed	Mean age (years)	Gender	Mean PANSS total score
Depressive Disorder	80	1.09%	26.87 (9.37)	Male (48.75%) Female (51.25%)	68.76 (17.96)
Bipolar Disorder	76	1.03%	25.05 (10.00)	Male (53.95%) Female (46.05%)	67.39 (16.17)
No Diagnoses provided	3	0.04%	Data unavailable	Male (66.67%) Female (33.33%)	79.00 (28.00)
PANSS Item Score missing	2	0.03%	Data Unavailable	Male (100.00%)	28.00 (1.41)
Total	161	2.19%	25.97 (9.57)	Male (52.17%) Female (47.83%)	68.30 (17.52)*

*Note*. N = 7,348; *n = 159, does not include two patients with missing PANSS item scores.

#### Data source

The data were provided by Ortho-McNeil Janssen Pharmaceuticals, Incorporated, and included a study identifier, de-identified patient number, gender, age at the time of study entry, age at the time of onset of illness, medication to which the patient was randomized, the patient's country of residence during the time of participation in the study and the scores for each of the 30 PANSS items for a baseline visit. In the interest of confidentiality, no treatment code information was included in the data, nor was there any exchange of information that might identify either the patients or the investigative sites taking part in the studies. The study was approved by the Institutional Review Board of Fordham University, New York.

#### Model Choice

Several key factors are involved in determining which model to use: (1) the number of item response categories, (2) the construct being measured, (3) the purpose of the study and, (4) the sample size [18]. Additionally, the nature of the construct being measured will affect the choice of the model.

**Figure 1:**
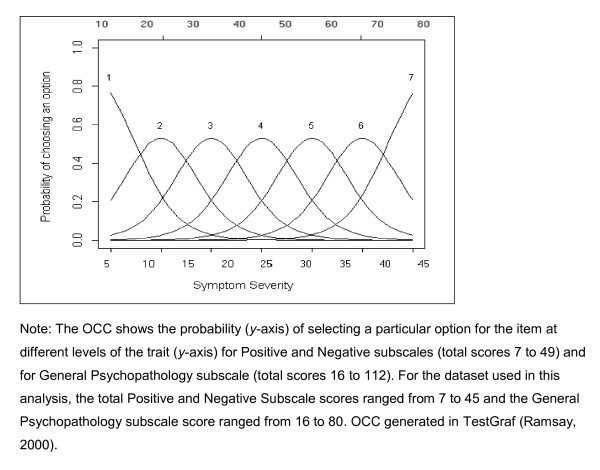
**OCC for a hypothetical "ideal"item**.

To investigate the usefulness of each item, the relationship between scores assigned to an item (i.e., the score ''option'' chosen for a given patient at a given point in time, such as 1to 7) and the overall severity of the illness (total subscale score) was assessed. For each item a set of Option Characteristic Curves (OCCs) is generated in which the probability of choosing a particular response is plotted against the range of psychopathology severity. OCCs are graphical representations of the probability of rating the different options for a given item across the range of severity. Using OCCs, the behaviour of particular items across a range of severity can be determined. If the probability of rating an option changes as a function of psychopathological severity, the option is useful; that is, it discriminates differences in illness severity. To illustrate, Figure 1 depicts a hypothetical "ideal" item from an item response perspective, which is characterized by a clear identification of the range of severity scores over which an option is most likely to be rated by a clinician (e.g., Figure 1 shows, option 1 is most likely to be rated from a score of 7 to a score of 20 on the Positive or Negative Symptoms subscale), rapid changes in the curves that correspond to changes in severity, and an orderly relationship between the weight assigned to the option and the region of severity over which an item is likely to be rated. An OCC, therefore, provides a graphical representation of how informative an item (or symptom) is as an indicator of the illness that is being measured, by expressing the probability of a particular option being rated by a clinician, at different levels of severity.

For the dataset used in this analysis, the total Positive and Negative Subscale scores ranged from 7 to 45 and the General Psychopathology subscale score ranged from 16 to 80. OCCs were generated in TestGraf [19].

Nonparametric IRT models [[20,21] and [22]] provide a broad-spectrum and flexible data analysis framework for investigating a set of polytomously scored items and determining ordinal scales for measurement that include items that have changeable locations and sufficient discrimination power [23].

IRT models are appropriate for the analysis of questionnaire data with multiple items [23] such as the PANSS. The data are discrete scores characterizing the ratings of *N *patients to J items (items are keyed *j*; *j *= 1,...*J*). Many measurement instruments, like the PANSS, use items that have three or more ordered answer categories characterized by three or more ordered scores, also called polytomous item scores.

#### Nonparametric IRT

A nonparametric Kernel Smoothing approach [24] to modelling responses for the PANSS would allow for no a priori expectation about the form of rating distributions, and items with nonmonotonic item response functions can be identified. Parametric and nonparametric approaches often lead to similar item selection [25]. Using a nonparametric approach, an ICC can be constructed that relate the likelihood of rating scores on each item to latent scores of psychopathology prior to examining the performance of individual options, and OCCs relate the likelihood of rating each option on each item to latent levels of psychopathology. Items' OCCs and ICCs can then be examined, and items with *weak *discrimination can be identified and can be considered for further item revision, or dropped from further analysis.

#### Approaches Used to Shorten Scales

Statistical methodologies used to shorten scales include simple correlations and adjusted correlations between long and short forms, Cronbach's *α *per dimension, item total correlation and item remainder correlation for item and composite scores, and factor analysis (see Coste et al [26] for review of methods used to shorten scales). A limitation of all these approaches is that the scores on the shortened scales are not comparable to the scores from the original scales, because they are not on the same metric.

#### Linking

Linking is a general term that refers to both equating and calibration. Whereas the requirements for equating are stringent, calibrating two assessments of different lengths is less so, and can easily be achieved using an IRT approach [27]. IRT is said to have a built-in linking mechanism [10]. Once item parameters are estimated for a population with an IRT model, one can calculate comparable scores on a given construct for patients from that population who were not rated on the same items, without intermediate equating steps. Previous examples of linking have been done with the PANSS supporting the extrapolation between PANSS and global clinical improvement and severity measures [28].

### Instruments

#### Positive and Negative Symptoms Scale

The PANSS [1] is a 30-item rating instrument evaluating the presence/absence and severity of Positive, Negative and General Psychopathology of Schizophrenia. All 30 items are rated on a 7-point scale (1 = absent; 7 = extreme). There are 3 subscales of the PANSS, the Positive Symptom subscale, the Negative Symptom subscale and the General Psychopathology subscale. The PANSS was developed with a comprehensive anchor system to improve the reliability of ratings. The 30 items are arranged as seven Positive subscale items (P1 - P7), seven Negative subscale items (N1 - N7), and 16 General Psychopathology items (G1 - G16). Each item has a definition and a basis for rating.

#### Rater Training

For the data being presented in this study, each PANSS rater, was required to obtain rater certification through Ortho-McNeil Janssen Pharmaceuticals, Incorporated, and to achieve interrater reliability with an intraclass correlation coefficient (95% *CI*) = 0.80 with the "Expert consensus PANSS" scores.

#### TestGraf

TestGraf software [19,24] was developed to estimate parameters in IRT [29]. TestGraf was used to estimate OCCs for nonparametric (Gaussian) smoothing kernels. This is a program for data analysis from tests, scales and questionnaires. In particular, it displays the performance of items and options within items, as well as other test diagnostics and utilizes nonparametric IRT techniques. Additionally, TestGraf provides a graphical analysis of test items and/or rated responses using Ramsay's "kernel smoothing" approach to IRT. The software, manual, and documentation are available from http://www.psych.mcgill.ca/faculty/ramsay/TestGraf.html[19].

### Procedure

TestGraf was used to fit the model. The highest expected total score produced by TestGraf is 45 for the Negative subscale, 40 for the Positive subscale. The General Psychopathology subscale had the highest expected total score of 80, at which the values of the OCCs were estimated. The estimation of the OCCs of the expected total score of the three PANSS subscales was made using a nonparametric (Gaussian) kernel smoothing technique [19,24] illustrated above. Examination of an item's OCC is expected to show how each response option contributed differently to the performance of that item [30]. The Item Characteristic Curves (ICCs) provides a graphical illustration of the expected score on a particular PANSS item as a function of overall psychopathology severity. ICCs were calculated in a similar manner as described above for OCCs. Items were characterized as "*Very Good"*, "*Good"*, or "*Weak*" based on the criteria presented in Table 2.

**Table 2:** Operational Criteria for Item Selection

Criterion and Ratings
Criterion 1. There is a range of severity in which the majority of items is expected to be more likely scored. This is represented by the number of options for which the item was more likely to be scored than all other options.
Basis of rating: Examination of the ICCs
Ratings for Criterion 1:
Yes: Items for which ≥ 5 options are selected will be considered for inclusion the abbreviated PANSS. The cut-off of four options was selected based on the median option score of PANSS (options range from one to seven).
No: Items for which ≤ 4 options are selected.

Criterion 2. The extent to which OCCs increase rapidly with changes in overall severity. Basis of rating: Examination of the OCCs.
Ratings for Criterion 2:
Yes: The probability (y-axis of OCC curve, see Figure 1) of selecting an option increases with increasing levels of severity. E.g. the probability of option 2 being selected doubles from 0.5 to 0.25 when severity increases from a score 18 to 12.
No: The probability (y-axis of OCC curve, see Figure 1) of selecting an option does not increase with increasing levels for all options. OCCs appear flat for each option.
Somewhat: The probability (y-axis of OCC curve, see Figure 1) of selecting an option increases with increasing levels of severity from the 50^th ^percentile of total score.

Criterion 3. The region in which each option is more likely to be selected is ordered, left to right, in accordance with their option scores on the OCC graphs.
Basis of rating: Examination of the OCCs.
Ratings for Criterion 3:
Yes: The severity regions and corresponding severity scores. E.g. the region in which option 2 is most likely to be selected (total severity score of 7 to 25 in Figure 1), falls between the regions in which option 1 (total severity score of 7 to 20 in Figure 1) and option 3 (total severity score of 7 to 35 in Figure 1) are most likely to be selected.
No: The severity region for which an option is most likely to be scored falls outside its two adjacent options for ≥ 5 options. E.g. the severity region in which option 3 is most likely to be scored ranges from a minimum score of seven to 35, and options 2 and 4 ranges from 10 to 30.
Somewhat: The severity region for which an option is most likely to be scored falls outside its two adjacent options for ≥ 1 and ≤ 4 options.

Criterion 4. Options for an item span the full continuum of severity from seven to 40 for the Positive subscale, seven to 45 for the Negative subscale, and 16 to 80 for the General Psychopathology subscale.
Basis of rating: Examination of the OCCs
Ratings for Criterion 4:
Yes: For a particular item, all seven options span the entire range of severity (from seven to 45 for the Positive and Negative subscales and 16 to 80 for the General Psychopathology subscale).
No: Greater than or equal to four options are more likely to be selected at higher (or lower) levels of severity than corresponding options. Four options were determined as the cut-off based on the median option score of the PANSS.

Criterion 5. As in parametric IRT, the slope or steepness of the curves indicates the item's ability to discriminate individuals along the latent continuum. In nonparametric IRT, the steeper a slope of the ICC, the more discriminant the item is. Slopes were computed in TestGraf for ICC graphs of each item from the median option score (i.e. four (Moderate) on the PANSS).
Basis of rating: Examination of the slopes ICCs.
Ratings for Criterion 5:
SYes: Slope is ≥ 0.40.
No: Slope is < 0.399

*Note*. An item was judged as *Very Good *if all of the five criteria were fulfilled, i.e. "Yes" for all five criteria. An item was judged as G*ood *if it has at least three of the five criteria, i.e. "Yes" for at least three criteria. An item was judged as *Weak *if it shows two or less of the five criteria. Items judged as *Weak *were removed from the original PANSS to constitute the abbreviated PANSS.

#### Operational Criteria for Item Selection

Using the ideal item illustrated in Figure 1, and following Santor and colleagues [8] operational criteria for item selection (numbers one to three presented below), items were judged on five criteria (see Table 2).

### Statistical Analyses

First, the complete dataset (n = 7,187) was randomly split into two subsamples, the Evaluation subsample (n = 3593) and the Validation subsample (n = 3594). All data were generated for this random sampling using SAS^® ^9.3.1 [31]. The Evaluation subsample and the Validation subsample were compared for similarities using t-tests for continuous variables and Chi-Square tests for categorical variables. The Evaluation subsample was used for the initial 30-item IRT.

A Principal Components Analysis (PCA) without rotation was conducted to assess unidimensionality as follows. A PCA without rotation was used as in general, an unrotated PCA is the best single summarizer of the linear relationship among all the variables, since rotated loadings may reflect an arbitrary decision to maximize some variables on a component while dramatically reducing others [32].: (1) a PCA was conducted on the seven Positive Symptom items, (2) the eigenvalues for the first and second component produced by the PCA were compared, (3) if the first eigenvalue is about three times larger than the second one, dimensionality was assumed. Suitability of the data for factor analysis was tested by Bartlett's Test of Sphericity [33] which should be significant, and the Kaiser-Meyer-Olkin (KMO) measure of sampling adequacy, which should be >0.6 [34,35].

Second, the criteria presented in Table 2 were examined. OCCs were used to examine Criteria 2, 3, and 4 presented in Table 2. For example, for Criteria 4, the options for an item are expected to span the full continuum of severity. Some options are expected to only be scored at high levels of severity (e.g., item G6 (Depression): options 6 and 7), whereas others are expected to be scored at low levels of severity (e.g., options 1 and 2). If the majority of options on an item are scored at only low levels of severity or only high levels of severity, that item was described as *Weak*. These items are considered *Weak *because they are difficult to score or do not contribute to the overall outcome and largely insensitive to individual differences in the lower or moderate range of symptom severity and produce floor effects. Scales comprised primarily of *Weak *items are also largely insensitive to individual differences in the high range of symptom severity and produce ceiling effects. Additional description of item selection is presented in Table 2.

Third, to confirm that most PANSS items are either *Very Good or *Good at assessing the overall severity, TestGraf program was used to produce the ICCs. The ICCs provided a graphical illustration of the expected total subscale score on a particular PANSS item as a function of overall psychopathology. ICCs were examined to assess Criteria 1 and 5 in Table 2. Finally, as in parametric IRT models, the slope or steepness of the curves indicate the item's ability to discriminate individuals along the latent continuum. Steeply increasing curves will indicate that the likelihood of higher item scores increases in close relation to increasing levels of psychopathology (V*ery Good *or G*ood *discrimination). Relatively flat curves or curves that do not show a consistent increasing linear trend indicate that the likelihood of higher item scores does not increase consistently as the level of psychopathology increases (*Weak *discrimination). The slope of the ICC was used to assess Criteria 5 presented in Table 2.

In nonparametric IRT, the steeper a slope, the more discriminant the item is. However, there are no specific statistical criteria to determine whether a slope is significantly steeper than another. The selection of a slope of 0.40 would allow for greater discrimination among items. In addition to the slopes, an item biserial correlation of items to expected total subscale scores was also produced for each item of the PANSS. TestGraf software produces slopes and item biserial correlations. It is expected that most items (i.e., > 60%) obtain a rating of *Very Good *or *Good *after examination of the OCCs, ICCs and item slopes for the operational criteria presented in Table 2.

Three graphs were used to determine the sensitivity to change for each subscale: (1) the average item information function graph, (2) the probability density function graph, and (3) the estimated standard error graph.

The average item information function was used to determine the amount of information in the test about severity, denoted by *I*(*θ*) This is produced in TestGraf and is a sum of item information functions [18,24].

A plot of the probability density function indicating the relative probability that various scores will occur was plotted to assess the score distribution of each subscale. The probability density function specifies how probable scores are by the height of the function, and the best-known example of a density function is the famous normal density, the "bell" curve.

Finally, for assessment of subscale performance, one of the most important applications of *I*(*θ*)was to estimate the standard error of an efficient estimate of *θ*, an efficient estimate being one which makes best use of the information in the PANSS subscales. also produced by TestGraf [18,24].

### Mini-PANSS

Using the IRT based methodology, an abbreviated version of the PANSS was created and scores were linked from the Mini-PANSS to the 30-item PANSS using an IRT summed score approach [16] and linear interpolation. Obtaining an IRT score, *θ*, corresponding to a summed score, rather than to a particular pattern of responses, requires finding the average of a posterior distribution.

Before linking the two scales, the unidimensionality of the Mini-PANSS was assessed using PCA without rotation, similar to the PCA conducted for the 30-item PANSS. Additionally, using the Validation subsample, Pearson correlation coefficients between total subscale scores on the 30-item scale and total subscale scores on the Mini- PANSS were computed. If the relationship between items (the item with item correlation is expected to be 1.0 as the items are rated by the same rater on the same patients) and subscales of the two instruments produce significant correlations (as identified by *p *≤ 0.001) given the overlap of items, this would suggest that the 30-item scale measures psychopathology similarly to the Mini-PANSS scale. A Cronbach *α*≥ 0.80 for each subscale and the total scale, are expected to show similarities between the PANSS and the Mini-PANSS.

We will be able to link the total score of the full scale PANSS, with very little bias, to scores on the "Mini PANSS" using a summed-score IRT based methodology and linear interpolation. It is expected that the 30-item PANSS and the Mini-PANSS show statistically significant correlations and a Cronbach *α *≥ 0.80 for each of the three subscales and total score, and that the differences in the 30-Item PANSS and interpolated scores on the Mini-PANSS have a small range of differences (≤ 5 points) with a mean error differences ≤ 1.

## Results

### Subsample Comparison

Comparison of the two sub-samples (Evaluation and Validation sample) by t-test for continuous variables and Chi-Square test,*χ*^2 ^for categorical variables across a range of characteristics, revealed no significant differences (Table 3).

**Table 3:** Comparison of Evaluation and Validation Subsample Characteristics

	Evaluation	Validation			Effect Size
Sample	3593	3594			
*Continuous *	Mean (sd)	Mean (sd)	t (7185)	p	Cohen's *d*
Age	39.49 (12.21)	39.43 (12.27)	0.048	0.962	0.005
PANSS					
Positive	19.43 (6.60)	19.57 (6.80)	- 0.961	0.373	- 0.021
Negative	22.70 (6.92)	22.71 (6.93)	- 0.049	0.961	- 0.001
General	40.82 (10.09)	40.85 (10.14)	- 0.142	0.887	- 0.003
Total Score	82.95 (19.30)	83.13 (19.60)	- 0.471	0.638	- 0.009
*Categorical *	n (Percent)	n (Percent)	Chi Square		
Gender					Cramer's*φ*
Male	2391 (66.55%)	2383 (66.30%)	*Χ*^2 ^(1) = 1.40	0.595	0.014
Female	1202 (33.45%)	1210 (33.67%)			
Diagnosis					
Schizophrenia	3437 (95.66%)	3429 (95.41%)	*Χ*^2 ^(1) = 1.04	0.669	0.012
Schizoaffective	156 (4.34%)	165 (4.59%)			
Race					
Caucasian	2407 (66.99%)	2432 (67.67%)	*Χ*^2 ^(4) = 13.70	0.134	0.044
Asian	255 (7.10%)	246 (6.84%)			
Black	614 (17.09%)	648 (18.03%)			
Hispanic	187 (5.20%)	177 (4.92%)			
Other	130 (3.62%)	91 (2.53%)			

*Note*. Level of significance, p ≤ .05 (two-tailed)

### Assessment of Unidimensionality

PCA without rotation revealed one component with an eigenvalue greater than one for the Positive Symptoms subscale, one component with an eigenvalue greater than one for the Negative Symptoms subscale and four components with an eigenvalue greater than one for the General Psychopathology subscale. Bartlett's Test of Sphericity was significant (*p *< .001) for all three subscales and the Kaiser-Meyer-Olkin (KMO) measure of sampling adequacy produced values of 0.789, 0.875, and 0.817 for the Positive, Negative and General Psychopathology subscales, respectively. Using the criteria to assess unidimensionality the Positive and Negative Symptoms subscales indicate unidimensionality while the General Psychopathology subscale shows an eigenvalue on the second component of only 1.915 times larger than the first component (see Table 4).

**Table 4:** Eigenvalues of PANSS subscale (without rotation)

PANSS Subscale	Component 1 Eigenvalue	Component 2 Eigenvalue	Ratio of Eigenvalues	Unidimensionality assumed
Positive Symptoms	3.172	.949	3.342	Yes
Negative Symptoms	3.867	.925	4.181	Yes
General Psychopathology	4.080	2.130	1.915	No

*Note*. Ratio of Eigenvalues is computed by dividing the eigenvalue for the first component by the eigenvalue for the second component for each subscale. Unidimensionality assumed is based on the eigenvalue of Component 1 being at least three times larger than the eigenvalue for Component 2.

### Positive Symptoms Subscale

#### Examining Option Characteristic Curves

**Figure 2:**
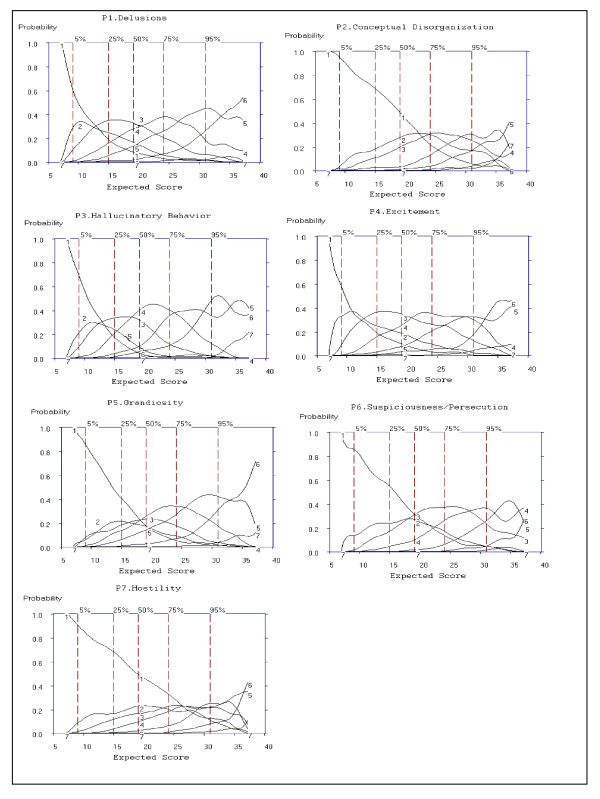
**Option Characteristic Curves (OCCs) for all 7 Positive Subscale Items of the PANSS**.

Figure 2 shows OCCs for items of the Positive Symptom subscale. The OCC for option 1 (absent) was less likely to be rated than other options for patients with higher severity scores. Option 7 (extreme) was rated infrequently, and the range of discrimination typically was above the 95th percentile (see Figure 2) indicating option 7 is most often rated for higher levels of severity. OCCs illustrated in Figure 2 were used to rate Criteria 2, 3, and 4 presented in Table 2.

For Criterion 2, the extent to which OCCs increase rapidly with change in overall severity was rated "Yes," "No" or "Somewhat" based on the probability (y-axis of Figure 2) of each option increasing as a function of overall severity. For items, P1, P3 and P4 the probability of rating options 1 to 7 increased as severity (x-axis: expected total score) increased and was rated as "Yes" for Criteria 2. Items P2, P5 and P6 show the probability of ratings from options 1 to 7 to increase rapidly primarily after the 50^th ^percentile of expected total score (i.e., an expected total score of 20), and were rated as "Somewhat" for Criterion 2. In the case of P7 the OCCs are flatter, even after the 50^th ^percentile. Therefore, based on Criterion 2 and examination of the OCCs, P7 (Hostility) does not increase rapidly with changes in overall severity, and was rated "No."

For Criterion 3, the severity region in which each option is more likely to be rated is ordered from left to right; the region in which option 2 is most likely to be scored should be between the regions in which option 1 and option 3 were scored. For P1, P2, P3, P4 and P6, option 2 always falls between the regions of options 1 and option 3. Additionally, option 3 falls between options 2 and 4, and option 4 falls between options 3 and 5. OCCs for P5 and P7 were rated as "Somewhat" on Criterion 3, as a visual examination of the curve shows that for P5, option 2 does not fall between options 1 and 3, and falls outside the curve of option 3 for lower severity scores. For P7, option 2 (expected total score of 7 to 37) does not fall between the regions in which option 1 (expected total score of 7 to 35) and option 3 (expected total score of 7 to 35) were scored, thereby being rated "Somewhat."

Based on Criteria 4, the options should span the full continuum of severity from expected total scores of seven to 40 for the Positive Symptoms subscale. An examination of the x-axis for expected total score shows all items were rated from a minimum total score of seven to the maximum total score for the Positive Symptoms subscale, and were rated as "Yes" for Criterion 4.

From examination of Figure 2 in comparison to the ideal item presented in Figure 1, it can be observed that items P1, P3, P4 and P6 most closely resemble the ideal item. Examination of the ICCs was combined with the results obtained for the OCCs to determine item selections for inclusion into the Mini-PANSS.

#### Examining Item Characteristic Curves (ICC)

For Criterion 1, items for which ≥ 5 options are scored would be rated "Yes." For items P1, P3 and P5 of the Positive Symptoms subscale, at least six options were selected (see y-axis to the highest point on the ICC). For example, for item P1, P3 and P5, the average item score climbs consistently as the total subscale score increases, approaching a maximum value of six out of the seven options. For P2, P4, P6 and P7, at least five options were selected. Therefore, for Criterion 1, a rating of "Yes" was given for all items. The cross-hatching or bars on the ICC indicates an estimated 95% confidence region for the true curve.

Criterion 5 of the operational criteria for item selection was identified in TestGraf by computing the slopes for each item from the median option choice of the PANSS (i.e. option 4). For further examination, items of the Positive Symptom subscale were ranked according to their numeric slope; the item with the largest slope was ranked Number 1, the item with the second largest slope was ranked Number 2, and so on. This rank ordering procedure was applied within the Evaluation subsample. For example, as shown in Table 5, item P3 was ranked first. Therefore, the item that was the most effective in discriminating individuals on the PANSS positive subscale was item P3, representing perceptions which are not generated by external stimuli. The last step in evaluating Criterion 5 was to determine the number of items for which the slope was ≥ 0.40. Slopes for P1 to P6 of the PANSS Positive Symptoms subscale were ≥ 0.40 and were rated "Yes" (see Table 5 for slopes).

**Table 5:** Slopes and Item Biserial Correlation for Each Item of the PANSS

Items	Slopes	Rank of Slopes	Item biserial correlation
P3. Hallucinatory behaviour	0.510	1	0.760
P5. Grandiosity	0.495	2	0.673
P1. Delusions	0.495	3	0.686
P4. Excitement	0.467	4	0.617
P2. Conceptual Disorganization	0.455	5	0.670
P6. Suspiciousness/Persecution	0.410	6	0.650
P7. Hostility	0.382	7	0.553

*Note*. Slopes were extracted from TestGraf output and were rounded to three decimal

places. Similarities in the values of slopes are due to rounding (e.g. slope of P1 is

0.495090, slope of P5 is 0.495447).

Table 5 also includes the biserial correlation for each item, or the correlation between a patient's score on an item (option 1 to option 7) and his or her expected total score on Positive Symptoms subscale. Although examination of the item biserial correlation is exploratory and not part of the operational criteria for item selection, the item biserial correlations show that item P7 has the lowest correlation compared to the other items of the Positive Symptoms subscale. The biserial correlations for the PANSS items ranged from 0.553 for item P7 to 0.760 for item P3.

#### Item Selection

**Figure 3:**
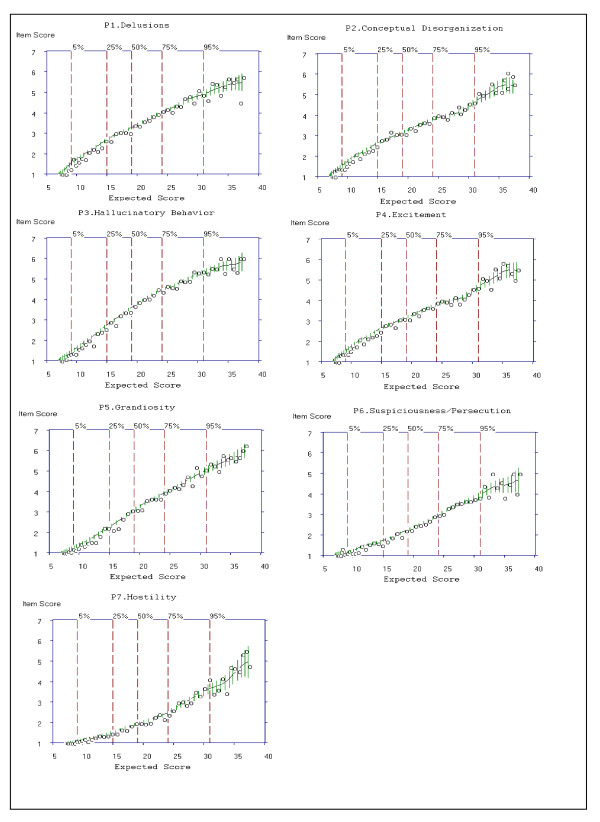
**Item Characteristic Curves (ICCs) for all 7 Items of the Positive Subscale of the PANSS**.

Global ratings of *Very Good*, *Good *and *Weak*, along with ratings of each of the five criteria for the seven items of the Positive Symptoms subscale are presented in Table 6 and were summarized above. Criterion 1 and 5 were based on examination of the ICCs presented in Figure 3. Criterion 2 to Criteria 4 was based on examination of the OCCs presented in Figure 2.

**Table 6:** Discrimination of the Positive Symptoms Items

Criterion	1	2	3	4	5	Rating
P1. Delusions	6 (Yes)	Yes	Yes	Yes	Yes	*Very Good*
P2. Conceptual Disorganization	5 (Yes)	Somewhat	Yes	Yes	Yes	*Very Good*
P3. Hallucinatory Behaviour	6 (Yes)	Yes	Yes	Yes	Yes	*Very good*
P4. Excitement	5 (Yes)	Yes	Yes	Yes	Yes	*Very Good*
P5. Grandiosity	6 (Yes)	Somewhat	Somewhat	Yes	Yes	*Good*
P6. Suspiciousness/ Persecution	5 (Yes)	Somewhat	Yes	Yes	Yes	*Very Good*
P7. Hostility	5 (Yes)	No	Somewhat	Yes	No	*Weak*

*Note*. Ratings are based on the operational criteria presented in Table 3.

### Negative Symptoms Subscale

#### Examining Option Characteristic Curves

**Figure 4:**
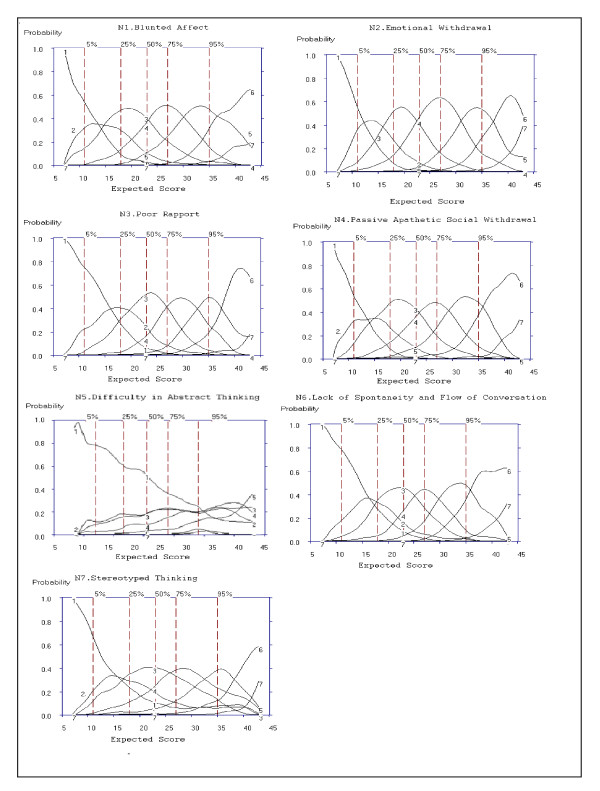
**Option Characteristic Curves (OCCs) for all 7 Negative Subscale Items of the PANSS**.

Figure 4 shows OCCs for items on the Negative Symptom subscale. Option 7 (extreme) was used infrequently; the range of discrimination was above the 95th percentile for all items, and only rated 0.1% of the time for N5 Difficulty in Abstract Thinking (Figure 4).

For Criterion 2, the extent to which OCCs increase rapidly with change in overall severity was rated "Yes," "No" or "Somewhat" based on the probability (y-axis of Figure 4) of each option increasing as a function of overall severity. For items, N1, N2, N3, N4, N6 and N7 the probability of rating options 1 to 7 increased as severity (x-axis: expected total score) increased and was rated as "Yes" for Criteria 2. In the case of N5 the OCCs are flatter, even after the 50^th ^percentile. Therefore, based on Criterion 2, N5 does not increase rapidly with changes in overall severity, and was rated "No."

For Criterion 3, the severity region in which each option is more likely to be rated is ordered from left to right; the region in which option 2 is most likely to be scored should be between the regions in which option 1 and option 3 were scored. For N1, N2, N3, N4, N6 and N7, option 2 always falls between the regions of options 1 and option 3, thereby attaining a rating of "Yes" for Criteria 3. Options for N5 were rated as "No" on Criterion 3, as a visual examination of the curve shows that for N5, option 2 does not fall between options 1 and 3, and follows the same pattern as option 3 from the 25^th ^percentile of the expected total score.

Based on Criteria 4, the options should span the full continuum of severity from expected total scores of seven to 45 for the Negative Symptoms subscale. An examination of the x-axis for expected total score shows that N1, N2, N3, N4, N5 and N7 were scored from a minimum total score of seven to the maximum total score for the Negative Symptoms subscale, and were rated as "Yes" for Criterion 4. An examination of the x-axis for the expected total score of N5 shows scoring started from a minimum expected total score of 10 and options 6 and 7 were only scored from an expected total score of 27 to 38. Additionally, Figure 4 shows that the probability of the OCC for item N5 is ≤ 0.3 for all options regardless of the level of severity.

#### Examining Item Characteristic Curves (ICC)

For Criterion 1, items for which ≥ 5 options are scored would be rated "Yes." For all items of the Negative Symptoms subscale, at least six options were selected (see y-axis to the highest point on the ICC). For example, for item N1, the average item score climbs consistently as the total subscale score increases, approaching a maximum value of six out of the seven options. Similar results are observed for all items of the Negative Symptoms subscale. Criterion 5 of the operational criteria for item selection was identified in TestGraf by computing the slopes for each item from the median option choice of the PANSS (i.e. option 4). For further examination, items of the Negative Symptom subscale were ranked according to their numeric slope; the item with the largest slope was ranked Number 1, the item with the second largest slope was ranked Number 2, and so on. The most effective in discriminating individuals on the PANSS was item N6, representing a reduction in normal flow of communication. The last step was to determine the number of items for which the slope was ≥ 0.40. Slopes for N1 to N6 of the PANSS Negative Symptoms subscale were ≥ 0.40 and were rated "Yes." Table 7 also includes the biserial correlation for each item and his or her expected total score on Negative Symptoms subscale. The item biserial correlations show the lowest correlations for item N5 (*r *= 0.599) and N7 (*r *= 0.596) compared to the other items of the Negative Symptoms subscale. The biserial correlations for the PANSS items ranged from 0.809 for item N2 to 0.596 for item N7.

**Table 7:** Slopes, Item Biserial Correlation for the Negative Symptoms subscale

Items	Slopes	Rank of Slopes	Item biserial correlation
N6. Lack of spontaneity and Flow of conversation	0.461	1	0.796
N1. Blunted Affect	0.447	2	0.771
N2. Emotional Withdrawal	0.447	3	0.809
N4. Passive Apathetic Social Withdrawal	0.447	4	0.773
N3. Poor Rapport	0.401	5	0.796
N5. Difficulty in Abstract Thinking	0.401	6	0.599
N7. Stereotyped Thinking	0.372	7	0.596

*Note*. Slopes were extracted from TestGraf output and were rounded to three decimal

places. Similarities in the values of slopes are due to rounding (e.g. slope of N1 is

0.447427, slope of N2 is 0.447313, slope of N4 is 0.447182, slope of N3 is 0.400801, and slope for N5 is 0.400560).

#### Item Selection

**Figure 5:**
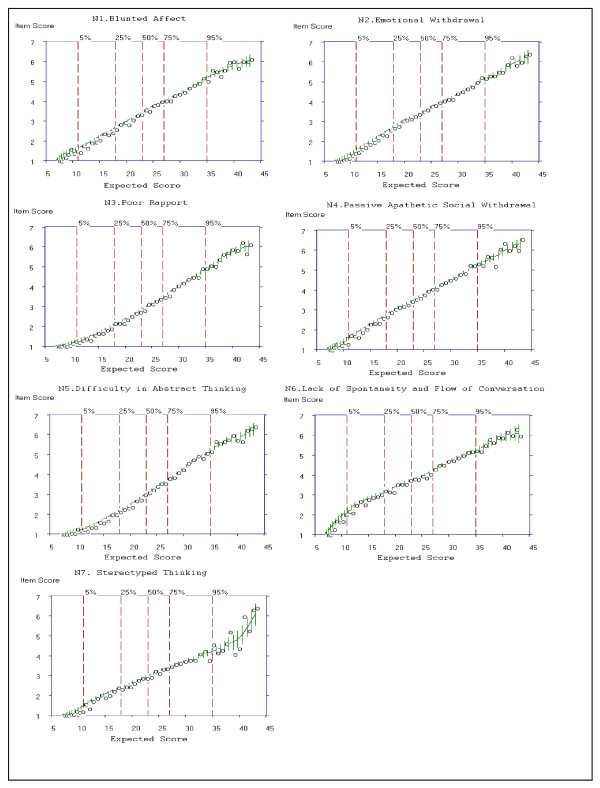
**Item Characteristic Curves (ICCs) for all 7 Items of the Negative Subscale of the PANSS**.

Global ratings of *Very Good*, *Good *and *Weak *items, along with ratings of each of the five criteria for the 7 items of the Negative Symptoms subscale were summarized above. Criterion 1 and 5 were based on examination of the ICCs presented in Figure 5. Criterion 2 to Criteria 4 was based on examination of the OCCs presented in Figure 4. Criterion 5 was based on examination of slopes (see Table 8 for item selections for the Negative Symptoms subscale).

**Table 8:** Discrimination of the PANSS Negative Symptoms Items

Criterion	1	2	3	4	5	Rating
Negative Symptoms						
N1. Blunted Affect	6 (Yes)	Yes	Yes	Yes	Yes	*Very Good*
N2. Emotional Withdrawal	6 (Yes)	Yes	Yes	Yes	Yes	*Very Good*
N3. Poor Rapport	6 (Yes)	Yes	Yes	Yes	Yes	*Very Good*
N4. Passive Apathetic Social Withdrawal	6 (Yes)	Yes	Yes	Yes	Yes	*Very Good*
N5. Difficulty in Abstract Thinking	6 (Yes)	No	No	No	Yes	*Weak*
N6. Lack of spontaneity/ Flow of conversation	6 (Yes)	Yes	Yes	Yes	Yes	*Very Good*
N7. Stereotyped Thinking	6 (Yes)	Yes	Yes	Yes	No	*Very Good*

*Note*. Ratings are based on operation criteria presented in Table 2.

### General Psychopathology Subscale

#### Examining Option Characteristic Curves

As observed with the Positive and Negative Symptoms subscales, the OCC for option 1 (absent) also were less likely to be rated than were other for patients with higher severity scores. Option 7 (extreme) was used infrequently; the range of discrimination was above the 95th percentile for all items.

**Figure 6:**
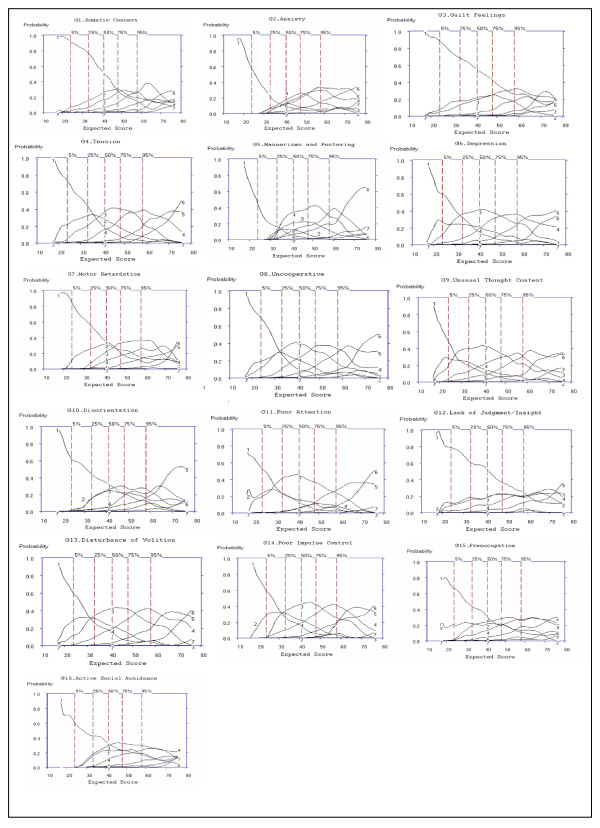
**Option Characteristic Curves (OCCs) for all 7 Negative Symptom Subscale Items of the PANSS**.

For Criterion 2, the extent to which OCCs increase rapidly with change in overall severity was rated "Yes," "No" or "Somewhat" based on the probability (y-axis of Figure 6) of each option increasing as a function of overall severity. For items, G4, G6, G7, G8, G9, G13 and G14 the probability of rating options 1 to 7 increased as severity (x-axis: expected total score) increased and was rated as "Yes" for Criteria 2. In the case of G1, G2, G3, G11, G12, G15 and G16, the OCCs are flatter, with G1, G2, and G3 showing increases only after the 75th percentile. Therefore, based on Criterion 2, G1, G2, G3, G11, G12, G15 and G16, does not increase rapidly with changes in overall severity, and was rated "No" for these items. A rating of "Somewhat" was given to G5 and G10 as these items show an increase in OCCs after the 50^th ^percentile. For example, an examination of item G12 shows that for options 1 to 7, the probability is ≤ 0.3 for the entire severity range, indicating this item does not discriminate between different levels of symptom severity. Similar probabilities (≤ 0.3 for across levels of severity) are seen for items G3, G15 and to a lesser extent G16.

For Criterion 3, the severity region in which each option is more likely to rated is ordered from left to right; the region in which option 2 is most likely to be scored should be between the regions in which option 1 and option 3 were scored. For G4, G6, G7, G8, G9, and G13, option 2 always falls between the regions of options 1 and option 3, thereby obtaining a rating of "Yes" for Criteria 3. Options for G3, G5, G10, G12, G15 and G16 were rated as "No" on Criterion 3, as a visual examination of the curve shows that for these items, option 2 does not fall between options 1 and 3, and in some cases (e.g. G10 and G12) follows the same pattern as option 3 from the 25^th ^percentile of the expected total score. OCCs for G1, G2, G11 and G14 were rated as "Somewhat" on Criterion 3, as a visual examination of the curve shows that for these items, option 2 does not fall between options 1 and 3, and falls outside the curve of option 3 for higher severity scores, however, for at least four other options, the severity region is ordered left to right, thereby being rated "Somewhat."

Based on Criteria 4, the options should span the full continuum of severity from expected total scores of 16 to 80 for the General Psychopathology subscale. An examination of the x-axis for expected total score shows that G4, G6, G8, G9, G10, G12, G13, G14, and G15 were scored from a minimum total score of 16 to the maximum total score for the General Psychopathology subscale, and were therefore rated as "Yes" for Criterion 4. An examination of the x-axis for the expected total score of G1, G2, G3, G5, G7, G11 and G16, shows scoring started from a minimum expected total score of 20 and for G3, options 4 to 7 were only scored from an expected total score of 36 to 76. For example, OCCs for Items G2, G5, and G16 do not span the continuum of possible total scores as scoring options 2 to 7 begin between the 5^th ^to 25^th ^percentiles. As a result, these items were rated "No" for Criterion 4. Results indicate that these items are only rated at higher levels of severity. It should be noted that seven out of the 16 General Psychopathology subscale items (43.75%) rated "Yes" on Criterion 2 (i.e., G4, G6, G7, G8, G9, G13 and G14), six out of the 16 subscale items (37.50%) rated "Yes" on Criterion 3 (i.e., G4, G6, G7, G8, G9, and G13), and nine out of the 16 subscale items (56.25%) rated "Yes" on Criterion 4 (i.e., G4, G6, G8, G9, G10, G12, G13, G14, and G15) based on examination of the OCCs.

#### Examining Item Characteristic Curves

**Figure 7:**
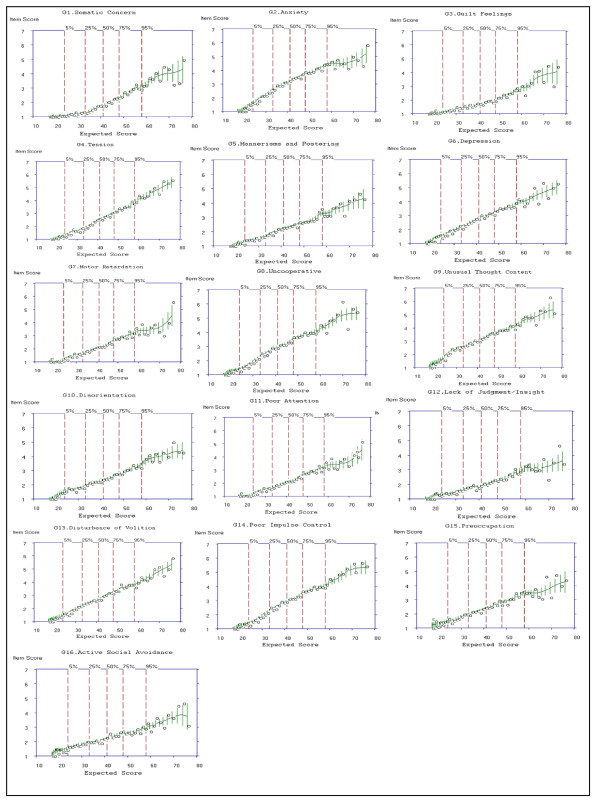
**Item Characteristic Curves (ICCs) for all 16 Items of the General Psychopathology Subscale of the PANSS**.

For Criterion 1, items for which ≥ 5 options are scored would be rated "Yes." For items G2, G4, G6, G7, G8, G9, G10, G14, and G15 of the General Psychopathology subscale, ≥ 5 options were selected (see y-axis to the highest point on the ICC, Figure 7); a rating of "Yes" was given for these items. Criterion 5 of the operational criteria for item selection identified nine items with slopes < 0.399; these items included G1, G7, G10, G3, G11, G15, G16, G5 and G12 in order of ranking of slopes and were rated "No" for Criterion 5. The item that was the most effective in discriminating individuals on the PANSS General Psychopathology was item G2, representing physical manifestations of nervousness, worry, apprehension or restlessness. The item least effective in discriminating individuals on the PANSS General Psychopathology subscale is G12, representing impaired understanding of one's psychiatric condition or illness (see Table 9 for slopes).

**Table 9:** Slopes and Item Biserial Correlation for General Psychopathology Subscale

Items	Slopes	Rank of Slopes	Item biserial correlation
G2.Anxiety	0.481	1	0.565
G14.Poor Impulse control	0.442	2	0.604
G4.Tension	0.437	3	0.630
G6.Depression	0.437	4	0.600
G9.Unusual Thought Content	0.437	5	0.605
G8.Uncooperative	0.431	6	0.617
G13.Disturbance of Volition	0.426	7	0.601
G1.Somatic Concern	0.395	8	0.541
G7.Motor Retardation	0.382	9	0.606
G10.Disorientation	0.382	10	0.441
G3.Guilt Feelings	0.377	11	0.431
G11.Poor Attention	0.377	12	0.567
G15.Preoccupation	0.377	13	0.438
G16.Active Social Avoidance	0.377	14	0.366
G5. Mannerisms and Posturing	0.373	15	0.463
G12.Lack of Judgment/Insight	0.350	16	0.381

*Note*. Slopes were extracted from TestGraf output and were rounded to three decimal

places. Similarities in the values of slopes are due to rounding (e.g. slope of G4 is

0.436681, slope of G6 is 0.436586, slope of G9 is 0.436546; slope of G7 is 0.381679, and slope for G10 is 0.381534; slopes for G3, G11, G15 and G16 are 0.377358, 0.377287, 0.377145, and 0.377003, respectively).

Table 9 shows item biserial correlations with the lowest correlations for item G16 (*r *= 0.366) and G12 (*r *= 0.381) compared to the other items of the General Psychopathology subscale. The largest biserial correlations was for item G8 (*r *= 0.617).

#### Item Selection

Global ratings of *Very Good*, *Good *and *Weak *items, along with ratings of each of the five criteria for the 16 items of the General Psychopathology subscale are presented in Table 10. Although the slope and item biserial correlation for G7 Motor Retardation is low, this item was retained for the Mini-PANSS as ≥ 3 criteria were scored "Yes" (see Table 10).

**Table 10:** Discrimination of the PANSS Items for General Psychopathology Subscale

Criterion	1	2	3	4	5	Rating
G1.Somatic Concern	4 (No)	No	Somewhat	No	No	*Weak*
G2.Anxiety	5 (Yes)	No	Somewhat	No	Yes	*Weak*
G3.Guilt Feelings	4 (No)	No	No	No	No	*Weak*
G4.Tension	6 (Yes)	Yes	Yes	Yes	Yes	*Very Good*
G5.Mannerisms/Posturing	4 (No)	Somewhat	No	No	No	*Weak*
G6.Depression	5 (Yes)	Yes	Yes	Yes	Yes	*Very Good*
G7.Motor Retardation	5 (Yes)	Yes	Yes	No	No	*Good*
G8.Uncooperative	5 (Yes)	Yes	Yes	Yes	Yes	*Very Good*
G9.Unusual Thought Content	6 (Yes)	Yes	Yes	Yes	Yes	*Very Good*
G10.Disorientation	4 (No)	Somewhat	No	Yes	No	*Weak*
G11.Poor Attention	4 (No)	No	Somewhat	No	No	*Weak*
G12.Lack of Judgment	4 (No)	No	No	Yes	No	*Weak*
G13.Disturbance Volition	5 (Yes)	Yes	Yes	Yes	Yes	*Very Good*
G14.Poor Impulse control	6 (Yes)	Yes	Somewhat	Yes	Yes	*Very Good*
G15.Preoccupation	4 (No)	No	No	Yes	No	*Weak*
G16. Social Avoidance	4	No	No	No	No	*Weak*

*Note*. Ratings are based on the operation criteria for item selection presented in Table 3.

Table 11 provides a summary of PANSS items which were rated *Very Good*, *Good *or *Weak *based on the operational criteria in Table 2. Nineteen items (63.33%) were rated as *Very Good *or *Good*.

**Table 11:** Summary of Items from the PANSS Identified as "Very Good," "Good," and "Weak."

Very Good Items	Good Items	Weak Items
P1. Delusions	P5. Grandiosity	P7. Hostility
P2. Conceptual Disorganization	G7. Motor Retardation	N5. Difficulty in Abstract Thinking
P3. Hallucinatory Behaviour		G1.Somatic Concern
P4. Excitement		G2.Anxiety
P6. Suspiciousness/Persecution		G3.Guilt Feelings
N1. Blunted Affect		G5.Mannerisms/Posturing
N2. Emotional Withdrawal		G10.Disorientation
N3. Poor Rapport		G11.Poor Attention
N4. Passive Apathetic Social Withdrawal		G12.Lack of Judgment
N6. Lack of Spontaneity		G15.Preoccupation
N7. Stereotyped Thinking		G16. Social Avoidance
G4.Tension		
G6.Depression		
G8.Uncooperative		
G9.Unusual Thought Content		
G13.Disturbance Volition		
G14.Poor Impulse control		

### PANSS Positive Symptoms Subscale Performance

**Figure 8:**
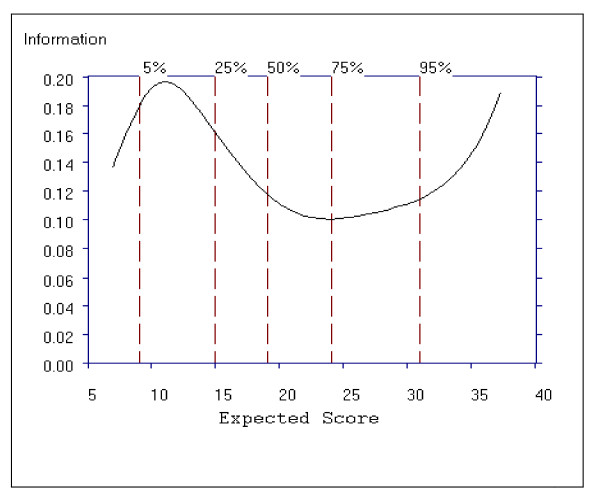
**Average Item Information Function for the Positive Symptom Subscale**.

Figure 8 shows the average item information function for the Positive subscale as a function of the total subscale score. For the positive subscale, the curve has one peak, around total score subscale scores of 8 to12, indicating that the scale is more informative for patients with lower scores, however the information function increases again after a total subscale score of 30, indicating the subscale contains discriminating items for patients with higher scores. Despite the peaks, the item information function is above 0.11 and below 0.20 with a difference of only 0.09 in information function.

**Figure 9:**
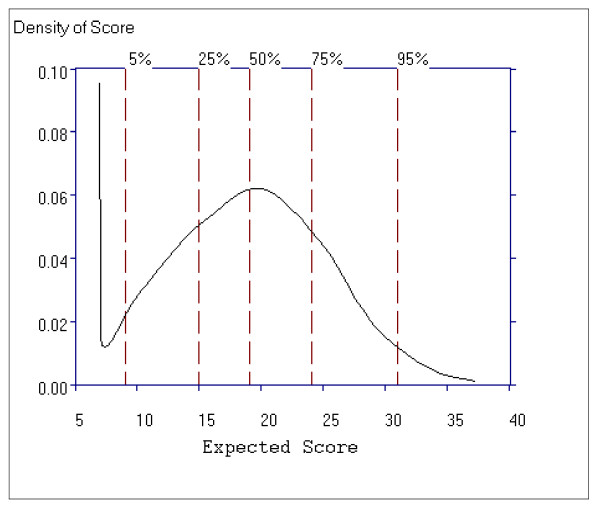
**The Probability Density Function for the Positive Symptoms Subscale**.

Figure 9 shows the distribution of Positive symptom scores and that this distribution is slightly skewed to the right, indicating that patients with very high Positive Symptoms subscale scores are rarer than patients with low subscale scores. The lowest score, a score of 7 (vertical line), and the scores in the 15 to 24 range are most probable.

**Figure 10:**
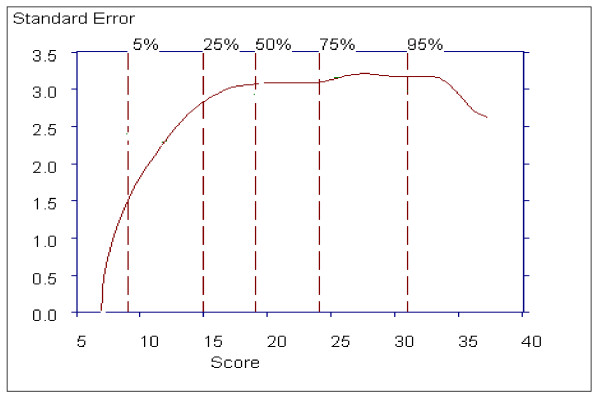
**Estimated Standard Error of the Positive Symptoms Subscale**.

Finally, Figure 10 shows that the standard error for a total subscale score of 17 to 34 is approximately 3.0, which includes 70% of patients. The standard error falls below 3.0 for patients scoring below 17 and above 34 possibly due to the poor quality of information in this subscale for patients with scores at the extremes of this subscale total score.

### PANSS Negative Symptoms Subscale Performance

**Figure 11:**
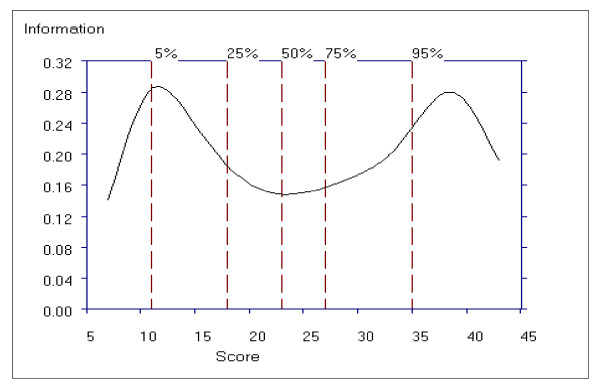
**Average Item Information Function for the Negative Symptom Subscale**.

Figure 11 shows the average item information function for the Negative Symptom subscale as a function of the total subscale score. For this subscale we see that the curve has two peaks, around the total subscale scores 9 to 13 and then again around the total subscale scores of 36 to 42, indicating that the subscale is more informative for patients with lower scores (9 to13) and higher scores (36 to 42). An item information function of greater than 0.14 and less than 0.29 on the theta scale is observed for scores on the Negative Symptom subscale.

**Figure 12:**
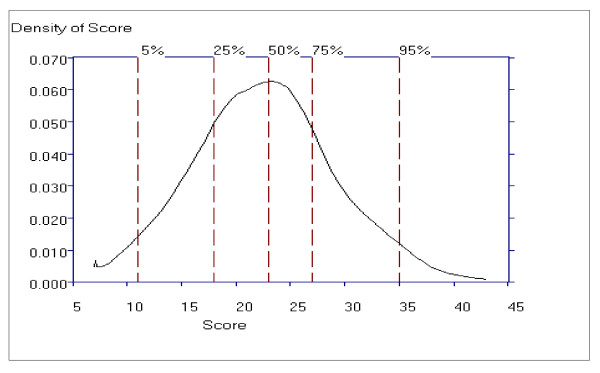
**The Probability Density Function for the Negative Symptoms Subscale**.

The probability density function for the Negative Symptom subscale shows the highest peak around scores in the 18 to 27 range indicating these score are the most probable, and that the probability trails off more gradually above this region than below, indicating positive skewness, and is a consequence of the rating relatively more higher scores than lower scores for this subscale (see Figure 12).

**Figure 13:**
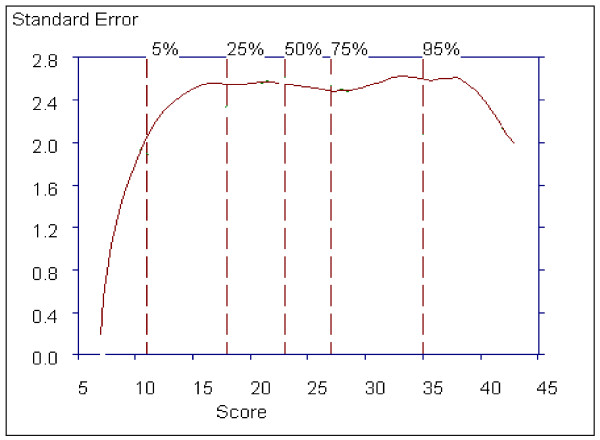
**Estimated Standard Error of the Negative Symptoms Subscale**.

Figure 13 shows the estimated standard error or sampling standard deviation of the total score as a function of severity. The standard error is approximately 2.4 for patients scoring in the range 15 to 37, which includes 80% of the patient scores. The standard error falls below 2.4 for patients scoring below 15 and above 37 possibly due to the poor quality of information in this subscale for patients with scores at the extremes.

### General Psychopathology Subscale Performance

**Figure 14:**
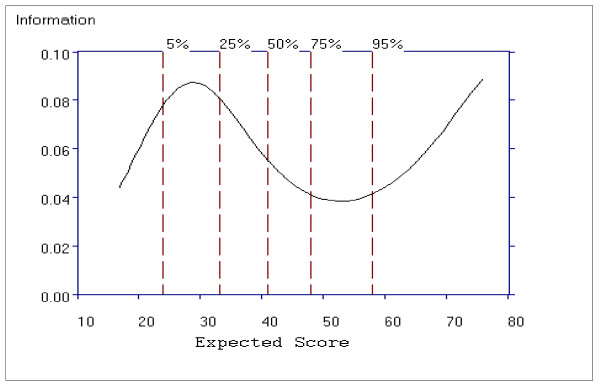
**Average Item Information Function for the General Psychopathology Subscale**.

Figure 14 shows the average item information function for the General Psychopathology subscale as a function of the total subscale score. For this subscale, the curve has one peak, around the scores ranging from 22 to 32, indicating that the scale is more informative for patients with lower scores, however the test information function increases again from a total score of 60, indicating the subscale contains many discriminating items for patients with higher scores. Compared to the range of information function for the Positive (0.20 to 0.09) and Negative (0.29 to 0.14) subscales, the information function for the General Psychopathology subscale shows a smaller range from approximately 0.04 to 0.09.

**Figure 15:**
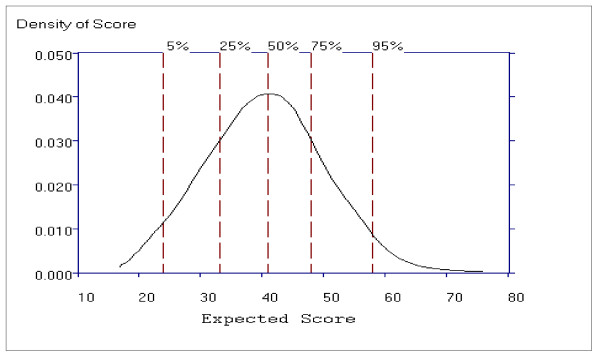
**The Probability Density Function for the General Psychopathology Subscale**.

Figure 15 shows the distribution of General Psychopathology subscale scores, which is slightly skewed to the right, indicating that patients with very high General Psychopathology subscale scores are rarer than patients with mid to low subscale total scores. The scores in the 32 to 48 range are most probable.

**Figure 16:**
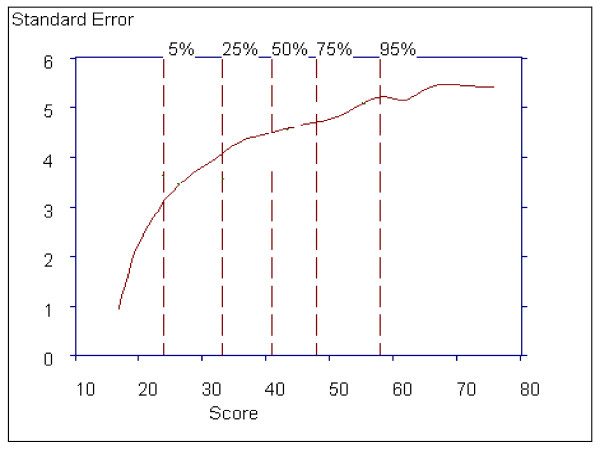
**Estimated Standard Error of the General Psychopathology Subscale**.

Figure 16 shows that the standard error for a total subscale score ranges from 1.0 to 5.0, and is above 3.0 for 95% of the sample. Smaller ranges of the standard error were observed for the Positive (< 0.01 to 3.0) and Negative subscales (0.2 to 2.4).

Examination of the item information function, probability density function and standard error of the PANSS subscales indicate that Positive and Negative subscales operate in a similar manner and are more discriminating than the General Psychopathology subscale scores, and may be more sensitive to change than the PANSS General Psychopathology subscale scores.

### Mini-PANSS

Based on the results of the nonparametric IRT presented above, 19 items were selected for inclusion in the Mini-PANSS. Only items selected, which were either *Very Good *or *Good *items (see Table 11). The Validation subsample (n = 3,494) was used to examine some of the psychometric characteristics of the 19 items selected for the Mini-PANSS. As a first step, a PCA without rotation similar to the PCA performed for the 30-item scale was conducted on the abbreviated 19-item PANSS to assess unidimensionality. Second, similarities between the two scales were examined by Pearson correlation coefficients between the 30-item scale and Mini-PANSS scale using the Validation subsample.

The mean PANSS scores of the Validation subsample for the 19-item PANSS are as follows, Positive subscale 17.52 (*SD *= 6.08) (6 to 36 score range), Negative subscale 19.01 (*SD *= 6.17) (6 to 39 score range), General Psychopathology subscale, 17.54 (*SD *= 4.85) (7 to 33 score range), and total PANSS 54.07 (*SD *= 13.27) (19 to 98 score range). Using the Validation subsample (n = 3,594), Bartlett's Test of Sphericity was significant (p < .001) for the six items of the Positive subscale, the six items of the Negative subscale, and the seven items of the General Psychopathology subscale. The Kaiser-Meyer-Olkin (KMO) measure of sampling adequacy produced values of 0.808, 0.858, and 0.804, for the Positive, Negative and General Psychopathology subscales, respectively. Using the criteria to assess unidimensionality of the eigenvalue for the first component being three times larger than the second component, the Positive, Negative and General Psychopathology subscales all indicate unidimensionality (see Table 12). The General Psychopathology subscale for the Mini-PANSS shows unidimensionality, compared to the assessment of unidimensionality for the 30-item PANSS (see Table 4) which did not show unidimensionality.

**Table 12:** Eigenvalues of Mini-PANSS subscales (without rotation)

PANSS Subscale	Component 1 Eigenvalue	Component 2 Eigenvalue	Ratio of Eigenvalues	Unidimensionality assumed
Positive Symptoms	2.920	.798	3.659	Yes
Negative Symptoms	3.628	.784	4.628	Yes
General Psychopathology	3.332	1.109	3.005	Yes

*Note*. Ratio of Eigenvalues is computed by dividing the eigenvalues for the first component by the eigenvalues for the second component. Unidimensionality assumed, is based on the eigenvalue of Component 1 being at least three times larger than the eigenvalue for Component 2 (as presented in the column titled 'Ratio of Eigenvalues')

Correlations were computed for the Positive, Negative, and General Psychopathology subscale scores, along with total PANSS scores for the 30 item PANSS and the Mini-PANSS. Significant correlations were observed between the respective subscale scores and the total scores of the two scales. Cronbach alpha *α*, between the 30-item PANSS and Mini-PANSS, ranged from .830 for the General Psychopathology subscale, .938 for the Positive Symptoms subscale, and .991 for the Negative Symptoms subscale suggesting that the subscales of the 30-item PANSS compared to the subscales of the Mini-PANSS all have high internal consistency as indicated by Cronbach's *α *≥ 0.80.

### Summed Score Linking

The item parameters from the 30-item scale and the item parameters from the 19-item scale were produced by TestGraf (expressed as, *θ *or IRT Score, ranging from - 3 to 3) and were used to estimate the IRT score corresponding to each summed score for each of the three subscales for the Validation subsample. After applying linear interpolation methods, Table 13, Table 14 and Table 15 display summed-score translation tables for the Positive Symptoms, Negative Symptoms and General Psychopathology subscales, respectively. IRT Score (*θ*) corresponding was converted to each expected total scores (summed score) for each subscale of the PANSS to the Mini-PANSS subscales prior to the application of linear interpolation (see Additional file 1, Table S1, Additional file 1, Table S2, Additional file 1, Table S3 for Conversion Table).

**Table 13:** Summed-Score Conversion Table for the Mini-PANSS and 30-item Positive Symptoms PANSS subscale based on expected total score

6 Item Positive Mini-PANSS	7 Item Positive Original PANSS	6 Item Positive Mini-PANSS	7 Item Positive Original PANSS
6	7	19	22
7	8	20	23
8	9	21	24
9	10	22	25
10	11	23	26
11	13	24	28
12	14	25	29
13	15	26	30
14	16	27	32
15	17	28	33
16	18	29	34
17	20	30	35
18	21	31	37

*Note*. Total scores on the Positive subscale for the 19-item Mini-PANSS are not presented for total scores > 32 based on expected total score ranges of the dataset used.

**Table 14:** Summed-Score Conversion Table for the 19-item and 30-item Negative Symptoms PANSS subscale based on expected total score.

6 Item Negative Mini-PANSS	7 Item Negative Original PANSS	6 Item Negative Mini-PANSS	7 Item Negative Original PANSS
6	7	22	26
7	8	23	27
8	9	24	28
9	10	25	29
10	12	26	30
11	13	27	32
12	14	28	33
13	15	29	34
14	16	30	35
15	18	31	36
16	19	32	37
17	20	33	38
18	21	34	39
19	22	35	40
20	24	36	42
21	25	37	43

*Note*. The 30-Item Negative subscale ranges from a total score of 7 to 49; the 19-item PANSS for the Negative subscale ranges from a score of 6 to 42. Total scores on the Negative subscale for the 19-item Mini-PANSS are not presented for total scores > 37 based on expected total score ranges of the dataset used.

**Table 15:** Summed-Score Conversion Table for the 19-item and 30-item General Psychopathology Symptoms PANSS based on expected total score.

7 Item General Mini-PANSS	16 Item General Original PANSS	7 Item General Mini-PANSS	16 Item General Original PANSS
7	19	20	45
8	23	21	47
9	25	22	49
10	27	23	51
11	29	24	52
12	30	25	54
13	33	26	56
14	34	27	58
15	36	28	60
16	38	29	62
17	40	30	64
18	41	31	66
19	43	32	67

*Note*. The 30-Item General Psychopathology subscale ranges from a total score of 16 to 112; the 19-item PANSS for the General Psychopathology subscale ranges from a score of 7 to 49. Total scores on the General Psychopathology subscale for the 19-item Mini-PANSS are not presented for total scores > 31 based on expected total score ranges of the dataset used.

As a final measure of comparison between the interpolation scores of the Mini-PANSS to the 30-item PANSS, the differences between the interpolated value from the Mini-PANSS and the actual score on the 30-item PANSS using the Validation subsample, was computed. For Positive Symptoms subscale, the mean difference between the interpolated score from the Mini-PANSS and the 30-item PANSS was -0.382 (- 4 to 3 range of scores). For the Negative Symptoms subscale, the mean difference between the interpolated score from the Mini-PANSS and the 30-item PANSS was -0.398 (- 4 to 4 range of scores). For the General Psychopathology subscale, the mean difference between the interpolated score from the Mini-PANSS and the 30-item PANSS was -0.407 (- 4 to 5 range of scores). For the PANSS total score, the mean difference between the interpolated score from the Mini-PANSS and the 30-item PANSS was -0.428 (-4 to 5 range of scores). The small mean differences and range of scores from the interpolated values support the similarities with the original scale.

## Discussion

The primary purpose of this study was to demonstrate that most of the items of the PANSS are *Very Good *or *Good *at assessing overall illness severity throughout the spectrum of increasing levels of severity. A second purpose was to create an abbreviated version of the PANSS using a nonparametric IRT in the TestGraf software. Shortened versions of this standardized and widely disseminated scale provide an interesting avenue, which could be more fully explored before investing resources in the development of completely new instruments.

Our results confirmed that a majority of PANSS items (63.33%; 19 out of 30 items) are either *Very Good *or *Good *at assessing the overall illness severity. Our results agree with the ones found by Santor and colleagues [8] who conducted the first IRT analysis of the PANSS. Not surprisingly our present nonparametric IRT showed that the Negative Symptom items (particularly, N1, N2, N3, N4, N6, and N7) showed good discriminative properties across almost the entire range of severity (i.e. increases in symptom intensity correspond to increases in illness severity), and it is these items that most closely approximates the "ideal" item illustrated in Figure 1. In addition, items of the Positive Symptoms subscale, P1, P3, P5, and to a lesser degree, P2, P4 and P6, also showed good approximation to the "ideal" item presented in Figure 1. For these items, the probability of rating a particular option (level of severity) corresponded to a relatively well defined and narrow range of severity.

In contrast, as demonstrated by Santor and colleagues [8] many items (P7, N5, N7, G1, G5, G6, G9, G10, G11, G12, G13, and G15) demonstrate problematic features and some fundamental issues remain with regard to the use of the PANSS total score as a measure of overall level of psychopathological severity in schizophrenia. Several items from the General Psychopathology subscale failed to show good discriminative properties across the range of severity assessed in the present study. Of the 16 items of the General psychopathology Subscale, only seven (43.75%) were found to be either Very Good or Good and were retained in the Mini-PANSS. For example, for item G3 (Guilt Feelings), OCCs were flat (not peaked) across almost the entire severity range, and was dominated by a single response option throughout most of the distribution of scores. One may argue that this is a result of the severity of the patient population used for this study, however, the levels of psychopathology in this study ranged from the lowest levels of severity (a total PANSS score of 32) to very high levels of severity (a total PANSS score of 161).

A consistent observation across all items was that very extreme symptomatology (option 7) was rarely rated. Additionally, Santor and colleagues [8] and Obermeier and colleagues [36] recommended rescaling the PANSS options as option 7 is rarely endorsed and some options present ambiguous definitions. For example, on item P1, patients scoring at the highest range of Positive Symptoms total score were far more likely to score a 5 or 6 on this item, suggesting that option 7 was underutilized. Additionally, a large number of items showed an overlap in OCCs for options 3 and 4 (some examples include G2, G3, G12). These result were not unexpected, because the definition of option 3 includes "little interference with patient's daily functioning,' whereas option 4 "represents a serious problem but occurs occasionally" (Kay et al., 1987). This phrasing appears to create greater overlap as the terms "little interference" and can be difficult to differentiate from "occurs occasionally." Results also demonstrate overlap between a number of adjacent OCCs. In particular, items P7, N5, G1, G3, G10, G11, and G12 display significant overlap between most options suggesting these levels of severity are poorly differentiated. Also, results show that some items are predominantly rated at higher levels of severity and do not span the entire continuum of expected scores. For example, G2, G5, and G16, have OCCs starting from expected scores on the General Psychopathology subscale of approximately 25.

It is noteworthy that results of the current investigation offered a high degree of agreement with other psychometric research of psychopathology in schizophrenia. Specifically, like the present IRT, previous psychometric investigations have indicated that PANSS items P7, N5, G1, G5, G10, G11, G12, G15 either do not discriminate well in terms of assessing overall severity or do not reflect dimensional individual differences between patients with schizophrenia [2,8,37]. Also, like the present IRT, previous psychometric investigations have indicated that PANSS items of N1, N2, N3, and N4 discriminate well and reflect dimensional individual differences [19,37,38]. The present results have implications for psychopathology measurement and clinical assessment. Researchers and clinicians evaluating psychopathology in schizophrenia using the 30-item PANSS may choose to focus only on items that performed well in IRT analyses.

The effectiveness of item options has a direct bearing on the effectiveness of their respective item and, therefore, on the effectiveness of the Positive, Negative, and General Psychopathology subscales. In this case, the Negative Symptom subscale was found to provide maximum information at the low and high ends of the construct. The low standard error of estimate supports the conclusion that these items form a well-defined subscale. Similar observations are noted for the Positive Symptom subscale with test information functions 0.10, and better for the lower 10% and upper 5% of the severity. The General Psychopathology subscale had the least test information function of the three subscales, ranging from 0.04 to 0.09 of the severity level. Additionally, standard error of estimate for the General Psychopathology subscale increased progressively from 1.0 at the lower end of the trait level up to 6.0 at the higher end of the severity level, thus indicating increased errors of measurement along higher levels of the severity continuum. These subscale performance results are similar to those found by Santor and colleagues [8], who observed better subscale performance for the Positive and Negative subscales over the General Psychopathology subscale. It appears then that the two subscale scores reflect the overall severity spectrum more appropriately than the total PANSS score. The use of the two Positive and Negative subscales independently from the rest of the scale is seen at times in clinical trials considering that these two symptom domains are key components of the disease [2] and which are primarily targeted in drug development.

Although the PANSS was originally designed with three subscales (Positive, Negative, and General Psychopathology), studies examining the internal structure of the scale [39] have all identified the same two underlying factors, a positive and negative factor. Other factors have varied and included Disorganized, Excitement, Hostility, Dysphoric, Catatonic and many more [2]. Given that OCCs depend on how symptom severity is defined, the appropriateness of modelling of items via their subscale scores, rather than a total PANSS score was confirmed by conducting PCA on each subscale to assess unidimensionality. The PCA of the General Psychopathology subscale did not assume unidimensionality, which supports to some extent the common practice in clinical trials to examine the Positive and Negative subscales independently from the rest of the scale since these symptoms are considered a key component of the disease [2] and are symptom clusters which are primarily targeted in drug development.

Our results of the nonparametric IRT provided valuable information regarding whether each item on the PANSS subscales was useful in the assessment of the overall severity of schizophrenia and in scale construct. In addition, it allowed us to select the PANSS items having utility across a broad range of illness severity and to include them in a shortened version of the scale (termed, Mini-PANSS). The similarities and differences between the 30-item PANSS and the Mini-PANSS were examined with a series of descriptive analyses, including high correlations between subscale and total scores. Results of the PCA of the Mini-PANSS assumed dimensionality for all three of the subscales. We deleted those PANSS items, which did not appear to contribute significantly to the symptom structure of schizophrenia based on their option curves. Exclusion of these less specific items (P7, N5, G1, G2, G3, G5, G10, G11, G12, G15 and G16) resulted in high internal reliability between PANSS 30-item subscales and Mini-PANSS subscales, indicating that omission of these items in future clinical trials is not likely to significantly alter the PANSS subscales. The performance of the Mini-PANSS relative to the original by comparing correlations and reliability of the 30-item PANSS subscales with the Mini-PANSS subscales was demonstrated by significant correlations and good reliability between the respective subscales, and the examination of the mean score differences between the interpolated scores and the actual PANSS scores show little bias in linking methods used.

This study illustrates a method of calibrating scales on the summed-score scale using an IRT approach. This method has been used in previous studies as the basis for the computation of IRT scaled scores for each summed score [16,40,41]. Although one may argue that some loss of information follows from the simplification of scoring from response patterns to summed scores, that loss of information is small and the corresponding change in the reported standard error would often not result in a visible change in the number of decimals usually reported.

We also developed a summed-score linking method to enable the transformation of the mini-PANSS scores for each of the subscales to the subscale scores of the full PANSS. This linking method will allow comparing data scored with the mini-PANSS to be transformed to the full PANSS allowing for comparison of results from studies using the two versions of the PANSS or to transform data from one study using the Mini-PANSS to data with the full PANSS. Future studies may benefit by incorporating a shortened version of the PANSS based on the items that performed as *Very Good *and *Good *in the IRT analyses. For example, abbreviating the measure in a meaningful way could serve as a screening instrument, increase rater reliability of assessment in research settings as well as offer an objective approach to measuring psychopathology in primary care and other clinical settings.

### Limitations

First, despite its advantage as a shorter instrument, the Mini-PANSS should not be considered as a replacement for the original scale. The decision to produce a short IRT- based form of the PANSS could be seen as a loss of the multidimensional construct. The PANSS dimensions of Anxiety/Depression, Excitement/Hostility, and Cognition are not fully represented in the Mini-PANSS. Even if a theoretical criterion was applied to select, among the most effective items, the different items that would eventually form a Mini-PANSS, one would need to re-examine these items from a theoretical perspective. Furthermore, there are still no definitive criteria to establish whether measures developed from IRT are theoretically and empirically superior to instruments developed with CTT.

Second, the present sample was based on patients included in clinical trials according to specific inclusion and exclusion criteria, and may therefore not accurately represent all patients with schizophrenia encountered in clinical practice and not be generalizable. Because of the large number of sites and investigators, interrater reliability among raters at different sites may not have been consistently optimal.

Third, our examination of OCCs showed that options in some items (e.g., item N5) were problematic, and that option 7 was rarely used at all levels of psychopathology. This may reflect the fact that patients included in clinical trials do usually not present with extreme levels of psychopathology. They could not be recruited and adequately consented at extreme levels of item severity. On the other hand, some adjustments may be necessary; for example, option 7 could be reformulated (e.g., combining options 6 and 7), and the effectiveness of these modifications will have to be empirically tested.

Fourth, Cella and Chang [42] warned of the possible limitations of using IRT methods in the evaluation of health measures since IRT methods were originally developed to be used with a fairly homogeneous educational assessment population. When we apply these methods to more heterogeneous clinical populations there may be limitations to obtain item-free estimates of sample latent traits. Cella and Chang [42] also remarked that the context, selection and sequence of items, considering both item diversity and clinical diversity, may produce sample-dependent item difficulty estimates and, therefore unreliable item-dependent estimates of patients' severity of illness. The continuous monitoring of item calibrations involved in the process of item banking will help to solve these uncertainties.

Finally, the full range of psychometric properties of the Mini-PANSS needs to be carefully studied before this new scale can be clinically used. We are presently planning to test these properties. For example, further examination of validity, reliability, sensitivity, specificity, schizophrenic categories and assessment of cut-off scores for the Mini-PANSS can be examined in a clinical trial framework.

## Conclusions

The primary purpose of this study was to demonstrate the utility of non-parametric IRT in examining the item properties of the 30 PANSS items and to select items for an abbreviated PANSS scale. We also provide a scoring algorithm for comparing total and subscale scores on the full scale to the total and subscale scores of the abbreviated scale. The comparisons between the 30-item PANSS and the Mini-PANSS revealed that the shorter version, when applying IRT, is also a better indicator of the latent trait, i.e. psychopathology severity.

One of the implications of our results is that some of the PANSS items need to be better defined in terms of item options and that it is possible to develop a shorter scale based on sound psychometric procedures. The availability of a Mini-PANSS will offer reduced administration time resulting in less clinician and patient burden during participation in clinical trials and in clinical practice. Future studies will focus on examining the psychometric properties of the mini-PANSS and on the improvement of some of the weaker PANSS items.

## Competing interests

### Financial competing interests

• In the past five years AK, CL or JPL have not you received reimbursements, fees, funding, or salary from an organization that may in any way gain or lose financially from the publication of this manuscript, either now or in the future.

• AK, CL or JPL do not hold any stocks or shares in an organization that may in any way gain or lose financially from the publication of this manuscript, either now or in the future.

• AK, CL or JPL do not hold or are currently applying for any patents relating to the content of the manuscript. AK, CL or JPL have not received reimbursements, fees, funding, or salary from an organization that holds or has applied for patents relating to the content of the manuscript.

• AK has received funding from Janssen Pharmaceuticals, LLP. JPL is a consultant for Janssen Pharmaceuticals, LLP and has received funding from National Institute of Mental Health, Astra Zeneca Pharmaceuticals, Pfizer Pharmaceuticals, Hoffman La Roche Pharmaceuticals, and Janssen Pharmaceuticals. CL has no competing funding interests.

### Non-financial competing interests

• AK, CL or JPL, has no non-financial competing interests (political, personal, religious, ideological, academic, intellectual, commercial or any other) to declare in relation to this manuscript.

## Authors' contributions

AK participated in the development of the concept for the study, design of the study, performed the statistical analysis and drafted the manuscript. CL assisted with the statistical analysis and helped draft the manuscript. JPL along with AK conceived of the study, and participated in its design and coordination and helped to draft the manuscript. All authors read and approved the final manuscript.

## Authors' information

AK obtained a degree in Psychometrics from Fordham University under the mentorship of CK, a statistician and Director of the Psychometrics Program at Fordham University, NY. AK has 8 years experience working in psychopharmacology research as a statistician and has peer reviewed publications in clinical trials in patients with schizophrenia, including collaborations on book chapters and journal articles with co-author JPL. AKs research interests are in Item Response Theory, Testing and Measurement and Bayesian applications in clinical research. CK studied at Princeton University, NJ, and is the current Director of Psychometrics Program at Fordham University, NY. CKs research interests are in Fairness and Validity in educational testing, Mental test theory, including item response theory and computerized adaptive testing, General(ized) linear models, including multiple comparisons and repeated measures, Bayesian inference, including multilevel modelling, Behavioural decision-making. CK has numerous publications in Item Response theory, Linear Modelling, Testing and Measurement, and Behavioural Decision Making. JPL is the Clinical Director at Manhattan Psychiatric Center and holds an academic position at New York University, NY. JPL is an expert on the PANSS Psychiatric rating scale and psychopharmacology research. He contributed to the development of a structured clinical interview for the PANSS and is involved in rater training for the PANSS. He has also published numerous factor analytic and psychometric studies on the PANSS and its use in clinical trials.

## Pre-publication history

The pre-publication history for this paper can be accessed here:

http://www.biomedcentral.com/1471-244X/11/178/prepub

## Supplementary Material

Additional file 1 **IRT Score (θ) to Expected Total Score Functions (Summed Score) prior to Linear Interpolation.** The file contains three conversion tables for IRT Score to Expected Total Score for Positive Symptom subscale of the Original PANSS and Mini-PANSS, IRT Score to Expected Total Score for Negative Symptom subscale of the Original PANSS and Mini-PANSS, and the IRT Score to Expected Total Score for Positive Symptom subscale of the Original PANSS and Mini-PANSS. Click here for file

## References

[B1] Kay SR Fiszbein A Lindenmayer JP Opler L Positive and negative syndromes in schizophrenia as a function of chronicity Acta Psychiatric Scandinavia 1986 74 507 518 10.1111/j.1600-0447.1986.tb06276.x 3492863

[B2] Van den Oord EJ Rujescu D Robles JR Giegling I Birrell C Bukszár J Factor structure and external validity of the PANSS revisited Schizop Research 2006 82 213 223 10.1016/j.schres.2005.09.002 16229988

[B3] Lindenmayer JP Harvey PJ Khan A Kirkpatrick B Schizophrenia: Measurements of Psychopathology Psychiatric Clinics of North America 2007 30 339 363 10.1016/j.psc.2007.04.005 17720027

[B4] Kay SR Opler LA Lindenmayer JP Reliability and validity of the positive and negative syndrome scale for schizophrenics Psych Res 1988 23 99 110 10.1016/0165-1781(88)90038-8 3363019

[B5] Peralta V Cuesta MJ Psychometric properties of the positive and negative syndrome scale (PANSS) in schizophrenia Psychiatry Research 1994 53 31 40 10.1016/0165-1781(94)90093-0 7991730

[B6] Lindenmayer JP Kay SR Friedman C Negative and positive schizophrenic syndromes after the acute phase: a prospective follow-up Comp Psychiatry 1986 27 276 286 10.1016/0010-440X(86)90003-9 3731764

[B7] Kay SR Fiszbein A Opler LA The positive and negative syndrome scale (PANSS) for schizophrenia Schiz Bulletin 1987 13 261 276 10.1093/schbul/13.2.261 3616518

[B8] Santor DA Ascher-Svanum H Lindenmayer JP Obenchain RL Item response analysis of the Positive and Negative Syndrome Scale BMC Psychiatry 2007 15 7 66 10.1186/1471-244X-7-66 PMC2211479 18005449

[B9] Nunnally JC Psychometric Theory 1978 2 New York: McGraw Hill

[B10] Embretson SE The new rules of measurement Psychol Assessment 1996 8 341 349

[B11] Hambleton RK Linn RL Principles and Selected Applications of Item Response Theory Educational Measurement 1989 New York: Macmillan 143 200

[B12] Lord FM Applications of Item Response Theory to Practical Testing Problems 1980 Hillsdale, NJ: Erlbaum

[B13] Van der Linden WJ Hambleton RK Handbook of modern item response theory 1997 Berlin: Springer

[B14] Santor DA Ramsay JO Progress in the technology of measurement: Applications of Item Response models Psych Assessment 1998 10 345 359

[B15] Bjorner JB Petersen MA Groenvold M Aaronson N Ahlner-Elmqvist M Arraras JI Use of item response theory to develop a shortened version of the EORTC QLQ-C30 emotional functioning scale Quality of Life Res 2004 13 1683 1697 10.1007/s11136-004-7866-x 15651539

[B16] Orlando M Sherbourne CD Thissen D Summed-score linking using item response theory: Application to depression measurement Psychol Assessment 2000 12 354 359 10.1037//1040-3590.12.3.354 11021160

[B17] Levine SZ Rabinowitz J Rizopoulos D Recommendations to improve the Positive and Negative Syndrome Scale (PANSS) based on item response theory Psychiatry Res 2011 15;188 3 446 52 21463902 10.1016/j.psychres.2011.03.014

[B18] Reeves BB Fayers P Fayers P, Hays R Applying item response theory modeling for evaluating questionnaire items and scale properties Assessing Quality of Life in Clinical Trials, New York 2005 2

[B19] Ramsay JO TESTGRAF A computer program for nonparametric analysis of testing data 2000 Unpublished manuscript, McGill University ftp://ego.psych.mcgill.ca/pub/ramsay/testgraf

[B20] Mokken RJ Linden WJvd & Hambleton RK Nonparametric models for dichotomous responses Handbook of modern item response theory 1997 New York: Springer 351 367

[B21] Petersen MA Book review: Introduction to nonparametric item response theory Quality of Life Research 2004 14 1201 1202

[B22] Sijtsma K Molenaar IW Introduction to nonparametric item response theory 2002 Thousand Oaks, CA: Sage

[B23] Lord FM Novick MR Statistical theories of mental test scores 1968 Reading MA: Addison-Welsley Publishing Company

[B24] Ramsay JO Kernel smoothing approaches to nonparametric item characteristic curve estimation Psychometrika 1991 56 611 630 10.1007/BF02294494

[B25] Lei PW Dunbar SB Kolen MJ A comparison of parametric and nonparametric approaches to item analysis for multiple choice tests Edu Psych Measurement 2004 64 565 587 10.1177/0013164403261760

[B26] Coste J Guillemin F Pouchot J Fermanian J Methodological approaches to shortening composite measurement scales J Clinical Epidemiology 2004 3 247 252 10.1016/s0895-4356(96)00363-0 9120523

[B27] Dorans NJ Linking scores from multiple health outcomes instrument Quality of Life Research 2007 16 85 94 10.1007/s11136-006-9155-3 17286198

[B28] Levine SZ Rabinowitz J Engel R Etschel E Leucht S Extrapolation between measures of symptom severity and change: an examination of the PANSS and CGI Schizophr Res 2008 98 318 22 10.1016/j.schres.2007.09.006 17949948

[B29] Patsula LN Gessaroli ME A comparison of item parameter estimates and ICC produced with TestGraf and BILOG under different test lengths and sample sizes 1995 Paper presented at the annual meeting of the National Council on Measurement in Education, San Francisco

[B30] Santor DA Coyne JC Examining symptom expression as a function of symptom severity: item performance on the Hamilton Rating Scale for Depression Psychol Assessment 2001 13 127 139 11281034

[B31] SAS Institute Inc SAS/STAT software: Changes and Enhancements through Release 9.1.3 2007 Cary, NC: Author

[B32] Smith EV Jr Smith EV, Smith RM Detecting and evaluating the impact of multidimensionality using item fit statistics and principal components analysis of residuals. Introduction to Rasch Measurement: Theory, Models and Applications 2004 Maple Grove, MN: JAM Press 12011501

[B33] Bartlett MS A note on multiplying factors for various chi square approximations J Royal Stat Society 1985 16 296 298

[B34] Kaiser HF A second-generation Little Jiffy Psychometrika 1970 35 401 415 10.1007/BF02291817

[B35] Kaiser HF An index of factorial simplicity Psychometrika 1974 39 31 36 10.1007/BF02291575

[B36] Obermeier M Schennach-Wolff R Meyer S Möller HJ Riedel M Krause D Seemüller F Is the PANSS used correctly? a systematic review BMC Psychiatry 2011 18 11 113 10.1186/1471-244X-11-113 PMC3146924 21767349

[B37] Kaiser HF A second-generation Little Jiffy Psychometrika 1970 35 401 415 10.1007/BF02291817

[B38] Fresan A De la Fuente-Sandoval C Loyzaga C Garcia-Anaya M Meyenberg N Nicolini H A forced five-dimensional factor analysis and concurrent validity of the Positive and Negative Syndrome Scale in Mexican schizophrenic patients Schizophrenia Research 2005 72 123 129 10.1016/j.schres.2004.03.021 15560957

[B39] Lindenmayer JP Bernstein-Hyman R Grochowski S Five factor model of schizophrenia: initial validation J Nervous Mental Disorders 1994 182 631 638 10.1097/00005053-199411000-00006 7964671

[B40] Thissen D Pommerich M Billeaud K Williams V Item response theory for scores on tests including polytomous items with ordered responses Applied Psychol Measurement 1995 19 39 49 10.1177/014662169501900105

[B41] Zeng L Kolen MJ An alternative approach for IRT observed-score equating of number-correct scores Applied Psychol Measurement 1995 19 231 240 10.1177/014662169501900302

[B42] Cella D Chang CH A discussion of item response theory and its applications in health status assessment Med Care 2000 38 1166 1172 10.1097/00005650-200009002-00010 10982091

