# Supplementary material for: Use of NON-PARAMETRIC Item Response Theory to develop a shortened version of the Positive and Negative Syndrome Scale (PANSS)
Source: BMC Psychiatry. 2011 Nov 16;11:178. doi: 10.1186/1471-244X-11-178 (PMC3256111; doi:10.1186/1471-244X-11-178)
Supplement: Additional file 1 — IRT Score (θ) to Expected Total Score Functions (Summed Score) prior to Linear Interpolation. The file contains three conversion tables for IRT Score to Expected Total Score for Positive Symptom subscale of the Original PANSS and Mini-PANSS, IRT Score to Expected Total Score for Negative Symptom subscale of the Original PANSS and Mini-PANSS, and the IRT Score to Expected Total Score for Positive Symptom subscale of the Original PANSS and Mini-PANSS. [file 1471-244X-11-178-S1.DOC]

ADDITIONAL FILE

**IRT Score (θ) to Expected Total Score Functions (Summed Score) prior to Linear**

**Interpolation**

Table S1

*IRT Score to Expected Total Score for Positive Symptom subscale of the Original PANSS and Mini-PANSS*

|  | PANSS | Mini-PANSS |  | PANSS | Mini-PANSS |
| --- | --- | --- | --- | --- | --- |
| IRT Score | 7 items | 6 items | IRT Score | 7 items | 6 items |
| -3 | 7 | 6 | 0.12 | 20.076 | 17.350 |
| -2.88 | 7.002 | 6.002 | 0.24 | 20.851 | 18.123 |
| -2.76 | 7.016 | 6.016 | 0.36 | 21.629 | 18.631 |
| -2.64 | 7.071 | 6.063 | 0.48 | 22.394 | 19.294 |
| -2.52 | 7.193 | 6.188 | 0.6 | 23.165 | 20.009 |
| -2.4 | 7.392 | 6.388 | 0.72 | 23.938 | 20.737 |
| -2.28 | 7.671 | 6.604 | 0.84 | 24.674 | 21.844 |
| -2.16 | 7.996 | 6.896 | 0.96 | 25.429 | 22.425 |
| -2.04 | 8.355 | 7.354 | 1.08 | 26.125 | 22.986 |
| -1.92 | 8.726 | 7.657 | 1.2 | 26.854 | 23.459 |
| -1.8 | 9.125 | 8.024 | 1.32 | 27.568 | 23.915 |
| -1.68 | 9.564 | 8.455 | 1.44 | 28.321 | 24.394 |
| -1.56 | 10.038 | 8.929 | 1.56 | 29.085 | 24.998 |
| -1.44 | 10.582 | 9.462 | 1.68 | 29.856 | 25.626 |
| -1.32 | 11.149 | 9.970 | 1.8 | 30.616 | 26.279 |
| -1.2 | 11.778 | 10.407 | 1.92 | 31.349 | 26.450 |
| -1.08 | 12.435 | 10.800 | 2.04 | 32.062 | 26.935 |
| -0.96 | 13.127 | 11.715 | 2.16 | 32.744 | 27.637 |
| -0.84 | 13.840 | 11.762 | 2.28 | 33.402 | 28.359 |
| -0.72 | 14.583 | 12.281 | 2.4 | 34.102 | 29.090 |
| -0.6 | 15.360 | 13.191 | 2.52 | 34.794 | 29.938 |
| -0.48 | 16.149 | 13.951 | 2.64 | 35.503 | 30.001 |
| -0.36 | 16.956 | 14.592 | 2.76 | 36.157 | 30.555 |
| -0.24 | 17.752 | 15.203 | 2.88 | 36.732 | 31.056 |
| -0.12 | 18.537 | 16.171 | 3 | 37.206 | 31.476 |
| 0 | 19.312 | 16.644 |  |  |  |

Table S2

*IRT Score to Expected Total Score for Negative Symptom subscale of the Original PANSS and Mini-PANSS*

|  | PANSS | Mini-PANSS |  | PANSS | Mini-PANSS |
| --- | --- | --- | --- | --- | --- |
| IRT Score | 7 items | 6 items | IRT Score | 7 items | 6 items |
| -3 | 7.092 | 6.089 | 0.13 | 29.085 | 24.776 |
| -2.99 | 7.256 | 6.250 | 0.26 | 29.856 | 25.366 |
| -2.86 | 7.542 | 6.422 | 0.39 | 30.623 | 26.123 |
| -2.73 | 7.934 | 6.765 | 0.52 | 31.349 | 26.836 |
| -2.6 | 8.384 | 7.178 | 0.65 | 31.859 | 27.283 |
| -2.47 | 8.865 | 7.631 | 0.78 | 32.259 | 27.659 |
| -2.34 | 9.379 | 8.071 | 0.91 | 32.963 | 28.284 |
| -2.21 | 9.919 | 8.571 | 1.04 | 34.008 | 29.129 |
| -2.08 | 10.508 | 9.017 | 1.17 | 34.960 | 30.060 |
| -1.95 | 11.755 | 10.082 | 1.3 | 35.497 | 30.530 |
| -1.82 | 11.802 | 10.133 | 1.43 | 36.114 | 31.111 |
| -1.69 | 12.539 | 10.749 | 1.56 | 36.622 | 31.523 |
| -1.56 | 13.533 | 11.612 | 1.69 | 37.307 | 32.18 |
| -1.43 | 14.212 | 12.193 | 1.82 | 38.555 | 33.129 |
| -1.3 | 15.621 | 13.395 | 1.95 | 39.818 | 34.69 |
| -1.17 | 17.470 | 14.974 | 2.08 | 40.834 | 35.432 |
| -1.04 | 18.674 | 15.706 | 2.21 | 40.902 | 35.335 |
| -0.91 | 20.235 | 17.164 | 2.34 | 41.140 | 35.538 |
| -0.78 | 22.019 | 18.903 | 2.47 | 42.077 | 36.410 |
| -0.65 | 24.355 | 20.539 | 2.6 | 42.192 | 36.306 |
| -0.52 | 25.429 | 21.435 | 2.73 | 42.910 | 36.913 |
| -0.39 | 26.125 | 22.123 | 2.86 | 43.273 | 37.168 |
| -0.26 | 27.198 | 23.189 | 2.99 | 43.769 | 37.569 |
| -0.13 | 27.706 | 23.598 | 3 | 43.986 | 37.777 |
| 0 | 28.321 | 24.115 |  |  |  |

Table S3

*IRT Score to Expected Total Score for General Psychopathology subscale of the Original PANSS and Mini-PANSS*

|  | PANSS | Mini-PANSS |  | PANSS | Mini-PANSS |
| --- | --- | --- | --- | --- | --- |
| IRT Score | 16 items | 7 items | IRT Score | 16 items | 7 items |
| -3 | 18.980 | 7.001 | 0.06 | 40.963 | 17.745 |
| -2.94 | 19.217 | 7.015 | 0.12 | 41.471 | 17.989 |
| -2.88 | 19.461 | 7.024 | 0.18 | 41.970 | 18.286 |
| -2.82 | 19.727 | 7.042 | 0.24 | 42.457 | 18.546 |
| -2.76 | 19.997 | 7.086 | 0.3 | 42.969 | 18.831 |
| -2.7 | 20.301 | 7.129 | 0.36 | 43.47 | 19.001 |
| -2.64 | 20.588 | 7.215 | 0.42 | 43.976 | 19.383 |
| -2.58 | 20.902 | 7.319 | 0.48 | 44.48 | 19.543 |
| -2.52 | 21.222 | 7.429 | 0.54 | 44.994 | 19.929 |
| -2.46 | 21.569 | 7.539 | 0.6 | 45.478 | 20.051 |
| -2.4 | 21.901 | 7.711 | 0.66 | 45.976 | 20.482 |
| -2.34 | 22.235 | 7.899 | 0.72 | 46.479 | 20.854 |
| -2.28 | 22.609 | 8.043 | 0.78 | 46.963 | 21.038 |
| -2.22 | 22.971 | 8.209 | 0.84 | 47.475 | 21.264 |
| -2.16 | 23.356 | 8.405 | 0.90 | 47.982 | 21.592 |
| -2.1 | 23.754 | 8.689 | 0.96 | 48.509 | 21.998 |
| -2.04 | 24.157 | 8.787 | 1.02 | 48.997 | 22.159 |
| -1.98 | 24.556 | 8.900 | 1.08 | 49.498 | 22.521 |
| -1.92 | 24.969 | 9.198 | 1.14 | 50.015 | 22.743 |
| -1.86 | 25.396 | 9.354 | 1.2 | 50.52 | 22.996 |
| -1.8 | 25.829 | 9.466 | 1.26 | 51.023 | 23.211 |
| -1.74 | 26.263 | 9.656 | 1.32 | 51.527 | 23.514 |
| -1.68 | 26.703 | 10.143 | 1.38 | 52.065 | 23.95 |
| -1.62 | 27.154 | 10.367 | 1.44 | 52.561 | 24.206 |
| -1.56 | 27.607 | 10.657 | 1.5 | 53.082 | 24.400 |
| -1.5 | 28.069 | 10.657 | 1.56 | 53.593 | 24.612 |
| -1.44 | 28.547 | 10.994 | 1.62 | 54.112 | 24.878 |
| -1.38 | 29.017 | 11.181 | 1.68 | 54.621 | 25.097 |
| -1.32 | 29.503 | 11.509 | 1.74 | 55.155 | 25.341 |
| -1.26 | 29.982 | 11.707 | 1.8 | 55.68 | 25.613 |
| -1.2 | 30.46 | 11.999 | 1.86 | 56.207 | 25.999 |
| -1.14 | 30.929 | 12.239 | 1.92 | 56.727 | 26.141 |
| -1.08 | 31.422 | 12.501 | 1.98 | 57.251 | 26.341 |
| -1.02 | 31.926 | 12.789 | 2.04 | 57.804 | 26.68 |
| -0.96 | 32.433 | 12.513 | 2.1 | 58.383 | 26.942 |
| -0.9 | 32.934 | 13.352 | 2.16 | 58.914 | 27.220 |
| -0.84 | 33.415 | 13.608 | 2.22 | 59.511 | 27.501 |
| -0.78 | 33.936 | 13.917 | 2.28 | 60.084 | 27.801 |
| -0.72 | 34.456 | 14.108 | 2.34 | 60.669 | 28.123 |
| -0.66 | 34.949 | 14.466 | 2.4 | 61.272 | 28.419 |
| -0.6 | 35.450 | 14.845 | 2.46 | 61.912 | 28.897 |
| -0.54 | 35.954 | 15.027 | 2.52 | 62.550 | 29.126 |
| -0.48 | 36.458 | 15.248 | 2.58 | 63.204 | 29.504 |
| -0.42 | 36.947 | 15.573 | 2.64 | 63.842 | 29.938 |
| -0.36 | 37.458 | 15.867 | 2.7 | 64.548 | 30.126 |
| -0.3 | 37.968 | 16.119 | 2.76 | 65.248 | 30.835 |
| -0.24 | 38.454 | 16.341 | 2.82 | 65.921 | 31.268 |
| -0.18 | 38.973 | 16.655 | 2.88 | 66.624 | 31.795 |
| -0.12 | 39.478 | 16.948 | 2.94 | 67.300 | 32.003 |
| -0.06 | 39.974 | 17.21 | 3 | 68.012 | 32.480 |
